# Economic factors influencing zoonotic disease dynamics: demand for poultry meat and seasonal transmission of avian influenza in Vietnam

**DOI:** 10.1038/s41598-017-06244-6

**Published:** 2017-07-19

**Authors:** Alexis Delabouglise, Marc Choisy, Thang D. Phan, Nicolas Antoine-Moussiaux, Marisa Peyre, Ton D. Vu, Dirk U. Pfeiffer, Guillaume Fournié

**Affiliations:** 1 Veterinary Epidemiology, Economics and Public Health Group, Department of Pathobiology and Population Sciences, Royal Veterinary College, University of London, Hawkshead Lane, Hatfield, Hertfordshire, AL97TA United Kingdom; 2 AGIRs-Animal and Integrated Risk Management Research Unit, CIRAD-Agricultural Research Center for International Development, Campus International de Baillarguet, Montpellier Cedex 5, 34398 Montpellier, France; 3 Wellcome Trust Major Overseas Programme, Oxford University Clinical Research Unit, 78 Giai Phong, Dong Da, Hanoi Vietnam; 4 MIVEGEC, University of Montpellier, CNRS 5290, IRD 224, 911 Avenue Agropolis, 64501, Montpellier cedex 5, 34394 France; 5 Center for Interdisciplinary Research on Rural Development, Vietnam National University of Agriculture, Ngo Xuan Quang Street, Trau Quy, Gia Lam, Hanoi Vietnam; 6 FARAH-Fundamental and Applied Research for Animals & Health, University of Liège, Avenue de Cureghem 7A-7D, Liège, 4000 Belgium; 7 School of Veterinary Medicine, City University of Hong Kong, 31 To Yuen Street, Kowloon, Hong Kong

## Abstract

While climate is often presented as a key factor influencing the seasonality of diseases, the importance of anthropogenic factors is less commonly evaluated. Using a combination of methods – wavelet analysis, economic analysis, statistical and disease transmission modelling – we aimed to explore the influence of climatic and economic factors on the seasonality of H5N1 Highly Pathogenic Avian Influenza in the domestic poultry population of Vietnam. We found that while climatic variables are associated with seasonal variation in the incidence of avian influenza outbreaks in the North of the country, this is not the case in the Centre and the South. In contrast, temporal patterns of H5N1 incidence are similar across these 3 regions: periods of high H5N1 incidence coincide with Lunar New Year festival, occurring in January-February, in the 3 climatic regions for 5 out of the 8 study years. Yet, daily poultry meat consumption drastically increases during Lunar New Year festival throughout the country. To meet this rise in demand, poultry production and trade are expected to peak around the festival period, promoting viral spread, which we demonstrated using a stochastic disease transmission model. This study illustrates the way in which economic factors may influence the dynamics of livestock pathogens.

## Introduction

The nature of the factors influencing the temporal dynamics of communicable diseases and, particularly, influenza viruses, are still subject to much discussion^1^. In most studies conducted on the epidemiology of human influenza viruses, climatic factors have been mentioned as major drivers of changes in disease incidence over time^2–4^. The temporal dynamics of zoonotic influenza viruses, which are transmitted from domestic animals to humans, have been comparatively less studied than those between humans, partly because their emergence as a public health concern is relatively recent. Examples of such viruses are H5N1 and H7N9 subtypes of influenza A, circulating in the domestic poultry populations of several countries in East, South and Southeast Asia, West Africa and Egypt^5, 6^. While transmission of avian influenza (AI) viruses to humans is limited and only occurs in case of close contact with infected birds or contaminated environment, the associated human case fatality rates are much higher than with human influenza viruses^7^. Therefore the risk that such viruses acquire the ability of inter-human transmission constitutes one of the major current threats to public health^8^. Some studies have pointed out the seasonal pattern of AI in endemic countries, with most cases occurring in November-March period^9, 10^.

So far most authors have used climate variables to explain this seasonality. Using Fourier transforms, a significant correlation between several climate variables (temperature, absolute and relative humidity and precipitations) and reports of H5N1 poultry outbreaks and human cases has been found in Egypt but not in Indonesia^11^. A link was found between annual precipitations and H5N1 incidence in China^12^. Spatial analyses conducted in South and Southeast Asia identified a correlation between domestic bird density and risk of H5N1 outbreak occurrence^13, 14^. In Thailand and Vietnam, the farming of free-grazing ducks in association with rice cultivation was shown to play a key role in the persistence and spread of the virus^14–16^ while a study in Bangladesh pointed that the presence of migratory birds’ staging areas, rivers and road used for trade and live bird markets were significant risk factors of H5N1 outbreaks^17^.

Field studies aimed at explaining the epidemiology of AI in Southeast Asia have suggested that trade of live poultry promotes potentially infectious contacts between poultry populations. A farm level case-control study has demonstrated that poultry farms with a high frequency of bird introduction or being located in the neighbourhood of poultry traders are at higher risk of AI infection^18^ while the potential role of live bird trade in AI propagation was also pointed out^19^.

Population density and intensity of transport of domestic animals are highly fluctuating in response to continuously changing economic conditions. Consumer demand for animal products, availability of inputs, change in technology or environmental hazards (e.g. livestock diseases) are among the main drivers of domestic animal population and trade dynamics^20^. The poultry production sector responds faster to demand changes than other animal production sectors, such as cattle and swine, due to its short production cycles^21^. Until now the potential impact of economic factors on poultry production and AI epidemiology have received little attention, mainly because of the lack of relevant data. Contrary to climatic variables, which are regularly recorded in meteorological stations, animal production and trade are not closely monitored, especially in developing countries, where these activities are mainly performed without official control and regulation.

Our study aimed to examine the influence of climatic and economic factors on the spatio-temporal distribution of outbreaks of Highly Pathogenic Avian Influenza type A subtype H5N1 (hereafter referred to as “avian influenza outbreaks” (AIO)) officially reported in the domestic poultry population in Vietnam. AIOs were defined as confirmed H5N1 AI cases having occurred in one or several neighbouring poultry farms or villages. The study period spanned from 2008 to 2015 and saw the report of 410 AIOs. Using Vietnam as a case study provides three advantages. First, there is a large climatic diversity in the country, from a semi-tropical climate in the North to a tropical climate in the South^2^. Second, this variation in climatic conditions does no correlate with the ethnic diversity of Vietnamese people, of whom the majority (89%) belongs to the *Kinh* ethnic group^22^. *Kinh* people are present across the whole country and share similar cultural features, including food consumption, religious customs and festivals. Finally, H5N1 AI has been endemic in the domestic poultry population of Vietnam since it was first reported in 2003, affecting southern as well as northern parts of the country^14^. The disease epidemiology is highly seasonal, with peaks occurring during the December-March season in all regions^23^. This seasonal pattern is still poorly understood.

The study was conducted in three successive stages: first, we investigated the influence of climate on the temporal variation of AI incidence by measuring the correlation between AI incidence and climate variables in regions of the country characterised by different climates. Then, in order to investigate whether other factors could explain AI seasonality, we used a finite mixture model to identify periods of time during which AIO frequency increased. Finally, we tested the influence of other factors using stochastic compartmental modelling.

## Results

### Identification of climatic regions

**Figure 1:**
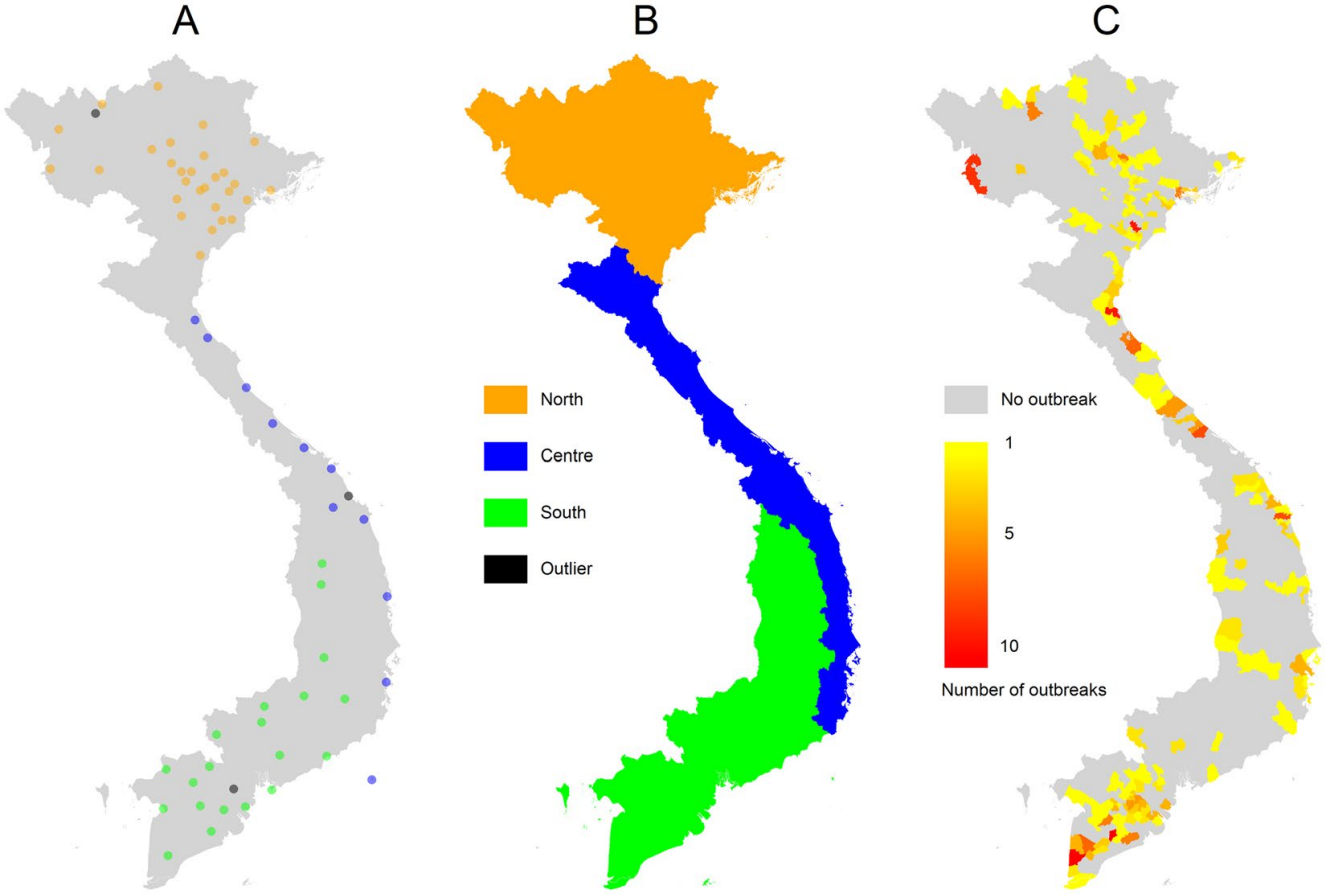
Selection of the three climatic regions of Vietnam. (**A**) Spatial location of weather stations belonging to each of the three identified clusters, differentiated by colour. (**B**) The three climatic regions. (**C**) Spatial location of AIOs reported in the domestic poultry of Vietnam from 2008 to 2015. Maps were produced using R 3.2.0 (http://www.R-project.org/). Administrative boundaries were drawn using GADM database of Global Administrative Areas, version 2.0 (www.gadm.org).

A preliminary step to the assessment of the correlation between AI incidence and climate variables was dividing the country into regions with homogeneous temporal variations of climate variables. The following climatic variables were considered: average relative humidity, average absolute humidity, cumulative rainfall, total number of hours of sunshine and average temperature. Using hierarchical clustering, 60 out of 63 Vietnamese weather stations were grouped into 3 clusters, comprising 29, 20 and 11 stations. An additional cluster of 3 outlier stations was discarded (Fig. 1A). Based on the spatial distribution and cluster membership of each station, we defined 3 climatic regions, later referred to as “North”, “Centre” and “South” (Fig. 1B). Reported AIOs were distributed as follows in these three regions over the study period (2008–15): North: n = 138 (33.6%); Centre: n = 113 (27.6%); South: n = 159 (38.8%) (Fig. 1C).

### Correlation between temporal variation of AI incidence and ecological variables

**Figure 2:**
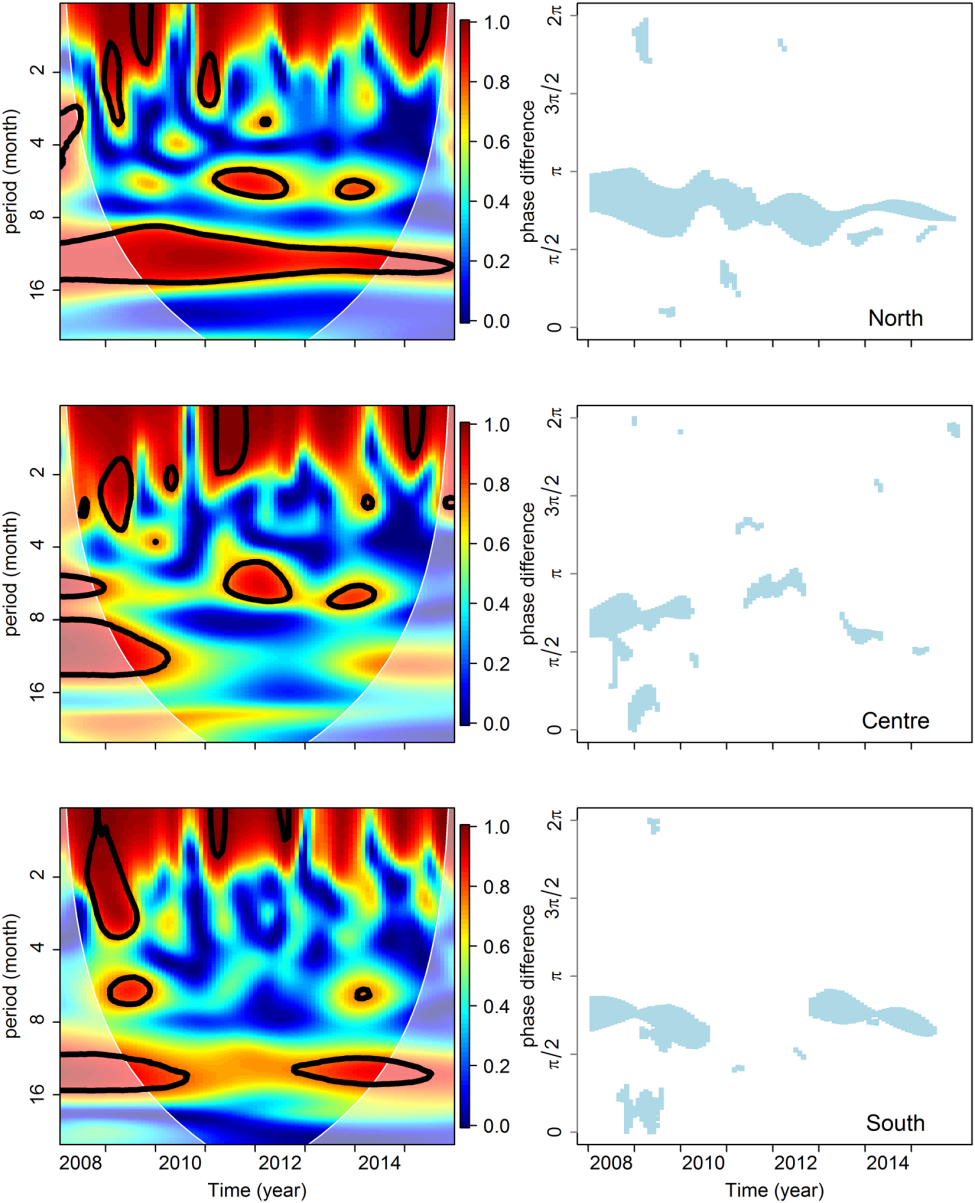
Wavelet coherence analysis between time series of reported AIOs and average absolute humidity in the three identified climatic regions of Vietnam (above: North, middle: Centre, below: South). Results of wavelet coherence analysis for the other climate variables are displayed in Supplementary Fig. S1. *Left:* Wavelet coherence indicated by a colour spectrum (blue: weak coherence, red: high coherence) as a function of the month of study period (x-axis) and the wavelet period (i.e. inverse frequency of wavelet oscillations) (y-axis). Black lines delineate areas of significant coherence between wavelet transforms (with an alpha risk ≤ 5%). White lines delineate the cone of influence, i.e. the area where computed coherences are strongly influenced by edge effects. *Right:* identified phase shifts from wavelet transforms of AI incidence to wavelet transforms of absolute humidity, when the two are significantly coherent.

In each identified climatic region, we computed the wavelet coherence between averaged monthly records of the 5 climatic variables and monthly counts of AIOs. Results obtained for the absolute humidity are displayed in Fig. 2 for illustration. Results for the other 4 climate variables are displayed in Supplementary Fig. S1. Coherences between all climate variables and monthly records of AIOs, for the one-year period (12 months), were significant (p value > 0.05) in the North region all along the study period. However, in the two other regions, coherences were not significant or not consistently significant over time (see for example Fig. 2A for absolute humidity).

Significant coherence between AIO incidence and absolute humidity at one year period was associated with a phase difference from AI incidence to absolute humidity comprised between $\pi /2$ and π (which correspond to a time difference of 3 to 6 months) in all regions (Fig. 2B). The same was observed for average temperature. For other variables, the phase difference varied with climatic region (Supplementary Fig. S1).

### Temporal clustering of AIOs

In order to develop an alternative hypothesis for explaining the temporal distribution of AIOs, we studied the distribution of AIO waiting times, i.e. the number of days between reporting dates of two successive AIOs. We aimed to identify underlying distributions of AIO waiting times, and seasonal periods during which certain durations of AIO waiting times are more frequent than others. We started by fitting finite mixture models of 1 to 4 exponential distributions to the distribution of AIO waiting times. In all climatic regions, mixtures of two exponential distributions resulted in the lowest Integrated Completed Likelihood (ICL) (ICL values are displayed in Supplementary Method S1), meaning AIO waiting times were most likely explained by mixtures of two exponential distributions. Therefore, AIO waiting times were clustered into two classes in all 3 regions: a class S including the shortest waiting times (North: n = 112; Centre: n = 86; South: n = 118), and a class L including the longest waiting times (North: n = 25; Centre: n = 26; South: n = 40).

**Figure 3:**
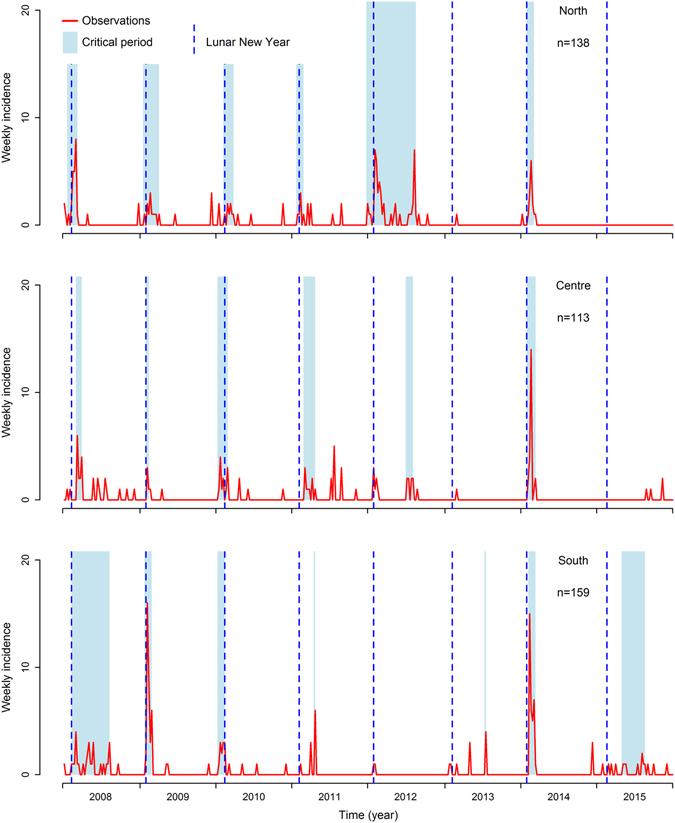
Weekly incidence of reported AIOs in the three climatic regions of Vietnam and estimated critical period and dates of Lunar New Year. n = number of AIOs reported in each of the climatic region during the study period.

Critical periods associated with the highest frequency of short AIO waiting times (class S) and the lowest frequency of long waiting times (class L) were identified for each year of the study period and each climatic region through an additional clustering process. The identified critical periods are displayed in Fig. 3. Most critical periods were narrow (1 to 66 days long), except in 2012 in the North and 2008 and 2015 in the South (>100 days) (Fig. 3). Overall, these critical periods only accounted for 14.9%, 6.5% and 12.7% of the study period in the North, Centre and South, respectively, while including the majority of AIOs (77.5%, 58.4% and 80.0% in the North, Centre and South respectively). A total of 10.6% of AIO waiting times were misclassified (i.e. attributed to critical periods while belonging to class L or vice versa) in the North (min: 6.0%; max: 25.0%), 20.6% in the Centre (min: 6.0%; max: 40.0%) and 12% in the South (min: 0.0%; max: 33.3%).

In 2008, 2009, 2010, 2011 and 2014 the beginning of the critical period coincided with or was close to the Lunar New Year festival, which took place at different dates from the 24^th^ January to the 20^th^ February (Fig. 3). It was not the case for 2012, 2013 and 2015 in which AIOs were either too few to allow clustering of their waiting times, or clusters were not related to the Lunar New Year.

### Seasonal variation in poultry consumption

The result exposed in the previous paragraph suggested that Lunar New Year celebrations were associated with higher AI incidence. We hypothesised that poultry meat consumption increased around the festival period. To meet this seasonal increase in demand, poultry density and trade activities intensified, thereby potentially promoting viral spread. In order to test this hypothesis, we first assessed whether poultry meat consumption significantly increased during traditional celebrations.

According to 2 inquired experts in poultry production, Vietnamese annual celebrations involving the consumption of poultry are Lunar New Year holidays (*Tết Nguyên Đán*), King Hung day (*Ngày Giỗ tổ Hùng Vương*), the fifth day of the fifth lunar month (only for duck meat) (*Mùng 5 tháng 5*) and the day of the dead (*Cúng cô hồn*). For some of the households, the reunification day or the independence day can also be an occasion for celebration. During these festivals, poultry meat may be used for traditional meals cooked for household consumption or worship of ancestors’ spirits. However, while most festivals take only one day, Lunar New Year celebrations take at least six days: one celebration on the eve of Lunar New Year (*Rước ông bà*), followed by celebrations during the first four days of the year (*Tân Niên*) and one celebration on the day trade activities restart after the holiday break (*Khai Trường Đầu Năm*). Festivities can extend well beyond before or after the Lunar New Year day, depending on households. In total, it was estimated that annual festivals involving the consumption of chickens took a minimum of 10 days (including 6 days during the Lunar New Year period) and a maximum of 22 days (including 17 days during the Lunar New Year period).

Based on data produced by the 2004 and 2012 Vietnamese Household Living Standard Survey (VHLSS), the ratio between the average daily quantity of poultry meat consumed during celebrations and the average daily quantity consumed during the remainder of the year were assessed (Table 1 **)**. The total quantity of consumed poultry meat increased 4.3 to 9.6 fold during the Lunar New Year period in 2004 and 2.9 to 6.6 fold in 2012. In both years, the increase in home-produced meat consumption was higher than the increase in purchased meat consumption, and the increase in chicken meat consumption was higher than the increase in the consumption of other poultry species (e.g. duck, quail or geese).

**Table 1:** Ratio between average daily poultry meat consumption during the Lunar New Year Period and outside that period in Vietnam (along with their 95% confidence intervals) by species and source (from Vietnam Households Livelihood Standard Survey in 2004 and 2012).

		2004		2012	
		Min (22 days of annual festivals)	Max (10 days of annual festivals)	Min (22 days of annual festivals)	Max (10 days of annual festivals)
Total		4.3 (4.1–4.5)	9.6 (9.1–10.1)	2.9 (2.8–3.1)	6.6 (6.3–6.9)
Species	Chicken	5.2 (5–5.5)	11.8 (11.2–12.3)	3.4 (3.3–3.6)	7.7 (7.3–8)
	Other species	2.6 (2.4–2.9)	5.9 (5.4–6.4)	2 (1.8–2.2)	4.4 (4–4.8)
Source	Purchased	3.8 (3.5–4.1)	8.6 (8–9.3)	2.6 (2.4–2.7)	5.8 (5.4–6.1)
	Home production	4.9 (4.5–5.3)	10.9 (10–11.8)	4.1 (3.6–4.5)	8.9 (8–9.8)
Climatic region	North	4.6 (4.3–4.9)	10.1 (9.5–10.8)	3.2 (3–3.4)	7.1 (6.6–7.6)
	Centre	6.3 (5.7–6.9)	14.3 (12.9–15.7)	4.7 (4.1–5.2)	10.5 (9.3–11.8)
	South	3.3 (3–3.6)	7.4 (6.8–8.1)	2.3 (2.1–2.5)	5.1 (4.7–5.5)

**Figure 4:**
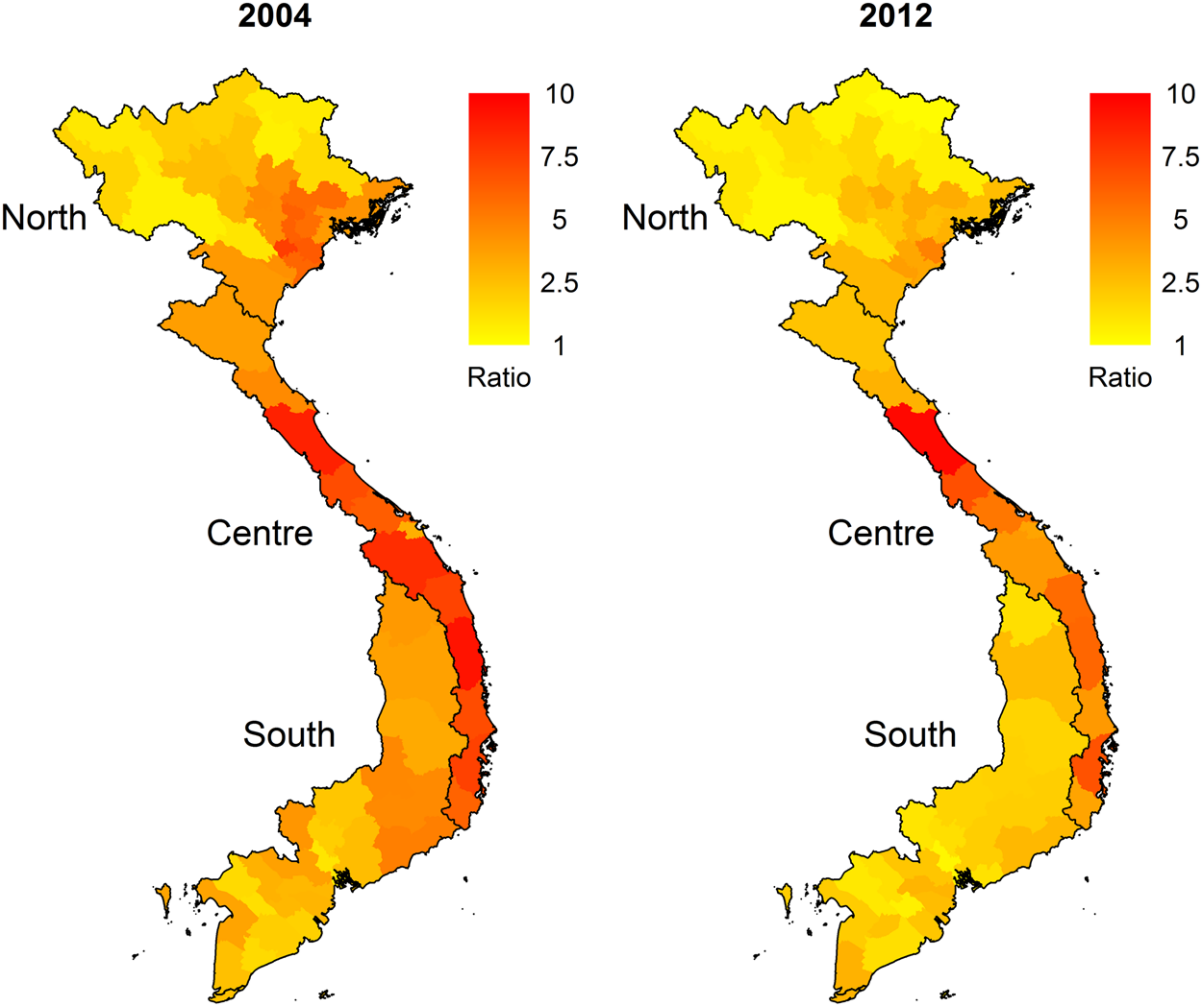
Ratio between average daily poultry consumption during the Lunar New Year festival period and outside that period for each province of Vietnam in 2006 and 2012. Black borders delimit the climatic regions identified through clustering. Maps were produced using R 3.2.0 (http://www.R-project.org/). Administrative boundaries were drawn using GADM database of Global Administrative Areas, version 2.0 (www.gadm.org).

In both years, there was a consistent increase in poultry consumption throughout Vietnam, but it was most pronounced in the Central part of the country (Fig. 4). The ratio was also lower in 2012 compared with 2004 in all climatic regions.

The average total poultry meat consumption per household was 1.47 times as high in 2012 as in 2004. The average quantity of poultry meat consumed by households during celebrations only increased by 9% from 2004 to 2012, while the quantity of poultry meat consumed by households during the rest of the year increased by 59% within the same period.

### The influence of seasonal variation in viral transmission on AIO patterns

We tested the hypothesis that the increase around the Lunar New Year period in the number of domestic birds produced and traded to fulfil the higher demand for poultry meat was associated with increased infectious contact rates between poultry farms. In contrast to climatic factors, the role of the Lunar New Year festival could not be addressed through correlation analysis, as no time series data on poultry production or consumption were available. Therefore, to test this alternative hypothesis, we used a stochastic Susceptible – Infectious – Recovered - Susceptible (SIRS) model to simulate AI transmission between Vietnamese poultry farms over time. We estimated the infectious contact rates outside and during the at-risk period (respectively noted *β*_0_ and *β*_*C*_) by fitting the model to the reported AIO time series for each climatic region, using an Approximate Bayesian Computation (ABC) algorithm. Other parameters were fixed. We assumed that the duration of this at-risk period (period during which the infectious contact rate *β*_*t*_ = *β*_*C*_) was 14 days (7 days prior to and after the Lunar New Year date, respectively).

The estimated ratios *β*_*C*_/*β*_0_ were highly variable and strongly influenced by the assumed duration of the infectious period (4 days or 13 days) (Table 2). However, for every set of parameters, the estimated infectious contact rate was always higher during the Lunar New Year period than during the remainder of the year (*β*_*C*_/*β*_0_ > 1) in all climatic regions. The 95% credible intervals of posterior *β*_*C*_/*β*_0_ of the three regions overlapped. Distributions of posterior ratios *β*_*C*_/*β*_0_ in the Centre and South regions were very close to each other, but the one in the North differed from those in the Centre and South, with a slightly lower median (Table 2). Posterior values of *β*_0_ and *β*_*C*_ are displayed in Supplementary Table S1.

**Table 2:** Ratios between inter-farm AI infectious contact rates during and outside the Lunar New Year period selected through Approximate Bayesian Computation (ABC) in the three climatic regions of Vietnam

Model parameters		Ratios of infectious contact rates (*β*_*C*_/*β*_0_*): median and 95% credible interval		
Duration of infectious period (days)**	Duration of removed period (days)***	North	Centre	South
4	15	2.9 (2.2–3.9)	3.4 (2.5–4.2)	3.5 (2.7–4.2)
	45	2.5 (1.8–3.4)	3.0 (2.1–3.7)	3.1 (2.3–3.7)
13	15	10.6 (5.8–20.1)	16.6 (9.7–23.0)	17.6 (11.2–23.3)
	45	9.4 (5.3–16.5)	14.6 (8.6–20.0)	15.5 (9.8–20.0)

*β_*C*_: Infectious contact rate during the at-risk period (Lunar New Year). *β*_0_: Infectious contact rate outside the at-risk period.

**Period from infection of farm birds to farm clearing.

***Period from farm clearing to repopulation.

**Figure 5:**
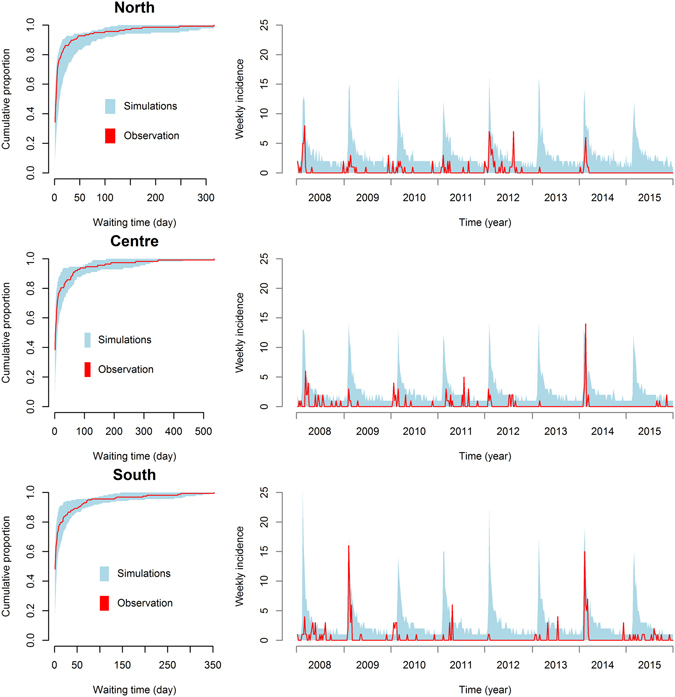
Results of the posterior predictive check in the 3 climatic regions of Vietnam. Observed and simulated cumulative distributions of AIO waiting times (Left) and observed and simulated AIO weekly incidence time series (Right). Parameters used: Duration of infectious period: 4 days; Time before repopulation: 15 days. Results obtained with other sets of fixed parameter values are displayed in Supplementary Fig. S2.

Results of posterior predictive checks are displayed in Table 3 and partly illustrated in Fig. 5, using only one set of fixed parameters. Results obtained with other parameter values are displayed in Supplementary Fig. S2. 100% of the observed cumulative distribution of AIO waiting times was contained within the minimum and maximum values simulated using the estimated *β*_0_ and *β*_*C*_ (Fig. 5) except for one set of parameters in the South (infectious period of 13 days and delay before repopulating the farm of 45 days) (Table 3). Pearson correlation coefficients between observed and simulated weekly AIOs counts were variable, but consistently higher than 0 in the 3 regions of the country, further suggesting that the model reproduced seasonal variations in AIO occurrence in these 3 regions (Table 3). For each set of fixed parameters, observed and simulated incidence peaks coincided, except in years 2013 and 2015 and the mid-2012 in the North and the Centre regions (Fig. 5).

**Table 3:** Results of posterior predictive checks in the three pre-defined climatic regions of Vietnam.

Model parameters		Proportion of the cumulative distributions of AIO waiting times accurately predicted by the model			Pearson correlation coefficient between observed and simulated weekly AIO incidence based on particles selections: median and 95% credible interval		
Duration of infectious period (days)*	Duration of removed period (days)**	North	Centre	South	North	Centre	South
4	15	1	1	1	0.4 (0.23–0.55)	0.24 (0.09–0.39)	0.37 (0.17–0.57)
	45	1	1	1	0.39 (0.21–0.56)	0.23 (0.09–0.42)	0.33 (0.12–0.57)
13	15	1	1	1	0.4 (0.22–0.54)	0.25 (0.13–0.42)	0.38 (0.19–0.56)
	45	1	1	0.98	0.39 (0.2–0.54)	0.24 (0.11–0.42)	0.36 (0.15–0.57)

^*^Period from the infection of farm birds to farm clearing.

**Period from farm clearing to repopulation.

## Discussion

In order to understand the influence of climate and poultry meat consumption on the temporal dynamics of AI in Vietnam, we applied different statistical and mathematical analysis methods to the data.

We first hypothesized that climate plays a significant role in the seasonality of AI, as already suggested by a previous study^9^. Wavelet coherence analysis showed a significant coherence between wavelet transforms of all climate variables and AIO time series in the North. In the Centre and the South, however, the coherence was not significant or not consistent all along the study period. The amplitude of intra-annual variation of temperature and absolute humidity was higher in the North compared with other parts of the country^2^, which might explain the higher values of wavelet coherence found in the North for these two variables. It was not the case, however, for precipitation, sunshine duration and humidity, which were highly variable in the central region as well. Therefore, while climate factors appeared to be potential explanatory factors of AI seasonality in the North, this was not the case for the Centre and the South.

On the other hand, the temporal distribution of AIOs in the 3 regions shared several characteristics as they were all most adequately explained by mixtures of 2 exponential distributions. Critical periods substantially associated with a shortening of AIO waiting times could then be defined. In 5 years out of 8, the occurrence of these critical periods was concomitant in the 3 regions and coincided with the Lunar New Year festival, in mid-January-mid-February.

We were able to estimate intra-annual changes in poultry meat consumption in Vietnam using the results of a national survey conducted with a large sample of households recruited throughout the country. The VHLSS survey did not record the duration of celebration periods. Therefore, durations of the Lunar New Year festival and other holidays during which Vietnamese *Kinh* households show a preference for poultry meat consumption were estimated based on the knowledge of experts in Vietnamese poultry production. The chosen range of possible values was sufficiently broad to ensure the validity of the results. Results differ between 2004 and 2012, the increase in daily consumption during celebrations being lower in 2012 compared with 2004. This is due to a 1.5 increase in poultry consumption outside festivities during the 2004–2012 period while the consumption during festivities remained nearly unchanged. Vietnamese households favour local chicken meat from native or crossed breeds for celebration purpose, whereas meat from industrial breeds is generally consumed outside celebration periods^24^. It is therefore reasonable to assume that the increase in the baseline poultry meat demand between 2004 and 2012 was partially or totally fulfilled by the increased imports of frozen industrial meat from abroad while the demand for poultry meat during Lunar New Year was fulfilled by the domestic sector^25^. Thus, the change in seasonality of poultry consumption did not necessarily have a strong effect on domestic poultry production. There were also spatial disparities, the increase in poultry consumption during the festivities being more pronounced in the Centre, less in the North and lowest in the South. This variability can be explained by slight cultural differences and economic development levels (the South and the Centre being the wealthiest and poorest regions of Vietnam, respectively^26^). Nevertheless, the amount of poultry meat consumed per day during the festival period was at least twice as high as during the rest of the year in the three regions in 2004 and 2012.

The estimated ratio between infectious contact rates during and outside the Lunar New Year period was consistently higher than 1 in all three climatic regions, regardless of the values of other model parameters. However, a precise estimate of this ratio was not possible, mainly because of the uncertainty associated with the values of other epidemiological parameters (duration of infection, time before repopulation, duration of period of increased trade). Although empirical quantitative data did not exist to validate our assumptions about these parameter values, the assumed ranges of possible values were kept sufficiently broad, while remaining realistic, to encompass the real values. This ensured the validity of our conclusion, the increase in the infectious contact rate during the Lunar New Year period.

Median ratios in the South and the Centre were very close each other, but median ratios in the North were slightly lower than in the two other regions for all pre-defined sets of parameters. Regional differences in surveillance sensitivity might explain this observation. National veterinary services, which produce the list of notified AIOs, have direct access to data on AI diagnostic testing performed by the North’s regional veterinary laboratory, but data on diagnostic testing performed in the Centre and the South have to be supplied by the regional veterinary services. As media reports of AI outbreaks involving larger numbers of flocks tend to increase subsequent notifications of AIOs^27^, it is possible that time series of AIOs supplied by the Centre and South regional veterinary services are biased toward times of high AI incidence, such as the Lunar New Year period, explaining the apparent higher infectious rate ratios found in these two regions.

As demonstrated by the posterior predictive check, for almost all sets of pre-defined parameters, estimated ratios were able to reproduce the distribution of the waiting times between successive reports of AIOs. In addition, time series of weekly incidence simulated with the estimated parameters were correlated with the observed incidence in the 3 climatic regions, showing that the effect of Lunar New Year was consistent across the whole country.

In China, animal trade has been shown to increase before Lunar New Year^28^. The same is likely to be true for Vietnam, as a logical consequence of the increase in poultry consumption. In particular, consumer preference for local chicken breeds during festivities means that a larger proportion of the consumed chicken comes from smaller-scale farms^24^. Buying and transporting poultry from a higher number of farms to the live bird markets and retail points requires a higher frequency of travel by traders and transporters and the mixing of birds from many different sources, promoting AI circulation. Alternatively, the number of poultry traders and transporters might increase before and during the Lunar New Year period, as people can easily endorse this activity which does not require a high initial investment^29^. Compared with regular traders, the experience and knowledge of newcomers in identifying and managing poultry health hazards is likely to be poor, placing them at higher risk of propagating AI.

The increased demand for poultry meat during the Lunar New Year period also may intensify illegal imports of live poultry from China^30^, potentially increasing the risk of infection of Vietnamese farms with foreign strains of AI viruses with varying levels of virulence^31^. This additional source of infection was not taken into account in the epidemiological model. However, according to a previous survey, this risk seems to be restricted to Northern Vietnam^30^.

The main limitation of the study is the quality of the AI surveillance data. Although the disease is considered to be endemic in Vietnam, only 410 AIOs were recorded during the study period which might actually be a small proportion of the true number of AIOs. The limited sensitivity of AI surveillance was previously pointed out^32^ and is attributed to several factors. These include the lack of incentives for farmers and other actors of the value chains to report AIO suspicions, misdiagnosis of Highly Pathogenic Avian Influenza as Newcastle disease, and the weak institutional links between local, regional and central veterinary services^27^. Moreover, the unit of an AIO was not always consistent. An “outbreak” generally was attributed to one farm, but in some cases it encompassed one or several villages. Despite the relatively small number of reported AIOs, their temporal distribution strongly departed from uniformity, due to the strong seasonal pattern of AI dynamics. While this data would not allow us to draw any quantitative conclusions about the true number of AIOs, they are still useful for exploring AI seasonality.

There may also be temporal biases in the reporting of AIOs. A survey showed that concerns of farmers, other actors of the poultry value chain and local veterinary agents over the poultry price drops associated with the notification of AIOs strongly limit their willingness to report AIO suspicions to the regional and central veterinary services. These drops in market prices are due to a temporary lowered demand of consumers for poultry and early sales of poultry by farmers, in fear that AI might infect their farms, causing a temporary high supply^27^. This concern might have a stronger effect in the months prior to Lunar New Year as a large number of farmers plan their production such that they are able to sell their flocks around the time of celebrations when they can achieve maximum profit. This is a possible explanation for the very low number of AIOs usually recorded in the few months preceding Lunar New Year.

There exist discrepancies between time series of AI incidence observed and predicted by our model. In addition to the regular peaks of AIOs observed in the Lunar New Year period, other peaks were recorded during summer 2012 in the North and the Centre, while no peaks occurred in 2013. Livestock market information provides possible explanations for these gaps. Several adverse events affected the Vietnamese husbandry sector in 2012, starting with a drop in demand for pig meat linked to public concern about the use of clenbuterol in hog finishing units, and an important epidemic of porcine respiratory and reproductive syndrome affecting successively the North, Centre and South Vietnam^33, 34^. In response, many pig farmers seem to have temporarily invested into poultry production^35^, which may have increased the number of poultry flocks and poultry sales, increasing the risk of H5N1 occurrence in the middle of 2012. The second event was a dramatic increase in swine and poultry commercial feed price which compelled many farmers to stop poultry farming and resulted in a supply deficit^33, 36^. This contraction of the poultry sector may be an explanation for the low number of AIOs in 2013. The deficit in poultry supply and resulting high sale prices during Lunar New Year 2013 encouraged the spontaneous launching of a high number of poultry flocks before Lunar New Year 2014^37^. This sudden increase in the poultry production and sales may also explain the high number of AIOs reported right after Lunar New Year 2014. Although this is a hypothetical explanation, it is highly plausible and consistent with the high adaptability of the Vietnamese livestock husbandry sector^24, 38^. It also emphasizes the importance of examining market data as potential explanatory factors of livestock contagious disease dynamics and for possible inconsistencies when comparing observed patterns with predictions produced by theoretical models.

From a veterinary and public health policy perspective, the results emphasize the need to better understand the influence of anthropogenic factors, and in particular economic drivers, on disease epidemiology. This could allow the identification of at-risk period for livestock and zoonotic diseases during which surveillance and control interventions (e.g. mass vaccination, sanitary inspection of transported animals) must be targeted. These results also suggest that economic variables, such as prices of animals’ products, could be included in models used to predict spatiotemporal variations in disease risk.

## Methods

### Selection of the study period

Although H5N1 AI was first reported in Vietnam at the end of 2003, only the 2008–2015 period was considered in this study. This was done since the temporal and spatial patterns in AIO reporting between 2004 and 2007 are likely to be linked to specific interventions: mass culling of infected farms, mass vaccination campaigns and ban of duck hatching^14, 23^. A total of 410 AIO were reported in Vietnam during 2008–2015.

### Data sources

Records of notified AIOs from January 2008 to December 2015 were provided by the Department of Animal Health of Vietnam. Records included dates of AIO detection (or date of reception of samples at the laboratory), and names of provinces and districts where AIOs were detected. Recorded AIOs were detected through passive surveillance which is based on the spontaneous report of AIO suspicions by poultry holders to local veterinary authorities. According to the Vietnamese government’s regulation, suspicions of AIOs were based on the observation of mortality or morbidity in domestic birds: death of 5% of a flock within 2 days or less, or mortality associated with some specific symptoms such as cyanosis or head swelling. AIOs were then confirmed through RT-PCR (Reverse transcription polymerase chain reaction) or virus isolation^39^.

Monthly meteorological data records from January 2008 to December 2015 were supplied by the Vietnamese Institute of Meteorology, Hydrology and Environment. These records came from 63 weather stations encompassing all provinces of Vietnam. Recorded variables were: average relative humidity (%), average absolute humidity (g/m^3^), cumulative rainfall (mm), total number of hours of sunshine and average temperature (°C).

Data about poultry consumption in Vietnam in 2004 and 2012 were supplied by the General Statistics Office of Vietnam. These data were collected through the Vietnamese Household Living Standard Survey (VHLSS) survey^40^. The 2004 VHLSS recruited 9189 households covering all provinces of the country, of which 9180 provided data on their poultry meat consumption. The 2012 VHLSS survey recruited 9399 households, all of which reported their poultry meat consumption. VHLSS questionnaires included questions on demographic characteristics of households as well as their sources of incomes and expenditures. Households were asked for the quantity of meat and eggs of chickens and other poultry species that they consumed during and outside holidays periods over the last 12 months, the quantity of poultry products that they produced at home and purchased, and the monetary value of these productions and purchases. The survey sampling was clustered and stratified by province and by type of area (urban and rural). Communes were randomly selected in each stratum; then 3 households per commune were randomly sampled. The survey was conducted all along the study year and each household was interviewed once^40^.

### Data availability

Files containing the data used in the study are available from the project registration “AI seasonality Vietnam” which can be accessed from: https://osf.io/93yvs/ (10.17605/OSF.IO/XMEYP).

### Identification of climatic regions

The objective of this section was to identify “climatic” regions of Vietnam with homogenous variation of climate variables over the study period (2008–2015). For this purpose, agglomerative hierarchical clustering was used to group 63 meteorological stations into clusters according to their level of similarity in monthly weather records of defined climatic variables^41^. Pearson correlation dissimilarities between time series from different stations were computed for each of the 5 variables separately and then averaged^42^. Ward’s minimum variance method was used for linkage^43^. Standardization was performed beforehand on each variable (i.e. subtraction of the mean and division by the standard deviation), to account for differences in magnitude between variables^42^. The number of clusters (i.e. climatic region) needed to be sufficiently large to account for the climatic diversity of Vietnam but, at the same time, each climatic region had to include a minimum of 10 meteorological stations, such that its area was large enough to account for a substantial proportion of reported AIOs. The optimal number of clusters satisfying these conditions was searched by successively trying 3 to 6 clusters. The clustering process was performed with the “HiClimR” R package^42^.

### Correlation between time variation of AI incidence and ecological variables

Correlation between monthly counts of AIOs and monthly averages of 5 climatic variables (average temperature, absolute humidity, relative humidity, sunshine duration, rainfall) in each of the identified climatic regions was assessed using wavelet coherence analysis^44, 45^, a tool particularly well adapted to deal with non-stationary time series such as epidemiological ones. Coherence between wavelet transforms of time series was calculated for each month of the study period and for different wavelet periods (varying from 1 to 25 months) using the “biwavelet” R package^46^. Statistical significance was assessed with a measure of coherence obtained through the generation of 2000 control time series with the same first order auto-regressive coefficient as the series of observations using Monte Carlo simulations^46^. Phase differences between wavelet transforms of significantly correlated time series were also obtained.

### Temporal clustering of AIOs

The objective of this section was to assess, in each identified climatic region, whether the observed distributions of AIO waiting times, i.e. the number of days between consecutive AIOs, resulted from a combination of several underlying distributions, and to assess whether these underlying distributions cluster in time.

First, underlying distributions were identified. Finite mixture models of 1 to 4 exponential distributions were fitted to the distribution of AIO waiting times^47^. Then the optimal number of components in the mixture model was selected on the basis of its Integrated Complete Likelihood (ICL)^48, 49^. Finally, each observed waiting time was assigned to the mixture component to which it was most likely to belong. The method is described in detail in Supplementary Method S1.

Next, we investigated whether the component distributions identified in the first step corresponded to different successive time periods of the year. For that we considered, in each year, a number of successive time periods equal to the number of component distributions identified in the first step, each time period corresponding to one of the components. The start and end dates of all the time periods were looked for by an exhaustive search using cross-entropy as the quantity to minimize^50^.

### Seasonal variation in poultry consumption

Here we aimed to assess whether the daily consumption of poultry meat by Vietnamese households changes during the Lunar New Year celebration period and to quantify this change. The VHLSS data collected in 2004 and 2012 were used to estimate the quantities of poultry meat consumed by households during and outside celebration periods. The VHLSS questionnaires include two separate sections on food consumption; one concerning the quantity of food consumed by interviewed households during annual celebrations, and another dedicated to food consumed during the remainder of the year. However, it does not indicate when these celebrations occur and their duration. Therefore, dates of annual celebration periods potentially involving the use of poultry meat were provided by two experts in poultry production from the National University of Agriculture of Hanoi. Based on this, a minimum and a maximum estimate of the number of days per year during which poultry meat is used for celebration purpose were calculated.

Using the summed durations as minimum and maximum denominators, we estimated the average quantity of poultry meat consumed daily by Vietnamese households during and outside the specified celebration periods as well as the ratio of the two means. In our calculation, we considered that only poultry meat consumption of households belonging to the *Kinh* ethnic group (89% of the population) increased during the mentioned celebration periods as minority ethnic peoples have their own traditional festivals at different times of the year. The sampling standard error and confidence interval of the ratio were estimated using first-order Taylor series expansion^51^. Weighting and clustering effects of the sampling design were accounted for in the calculation of the standard error, and sample weights were accounted for in the calculation of the ratio^52^.

### The influence of periods of high consumption on viral transmission and AIO distribution

Here the aim was to assess whether the infectious contact rate between poultry farms increased during periods of the year characterized by increased poultry trade in response to a higher poultry meat demand.

A stochastic compartmental model was used to simulate the between-farm transmission of AI in Vietnam from 2008 to 2015. The epidemiological units of the model were poultry farms, which could pass through 3 infection states: susceptible (i.e. only composed of susceptible birds), infectious (i.e. composed of at least one infectious bird) and removed (i.e. empty). The transition from the susceptible to the infectious state was caused by the infection of at least one bird on the farm through contact with an infectious farm (e.g. due to movement of infectious birds or contaminated fomites). The spread of infection within a farm was not explicitly modelled and the infectiousness of a farm was assumed to remain constant throughout its infectious period. The transition from the infectious to the removed state was caused by the depopulation of the poultry flock, i.e. the destruction or sale of the birds. The transition from the removed to the susceptible state was caused by the re-introduction of susceptible birds in the farming unit. The number *N* of farms remained constant over time. The infectious contact rate *β*_*t*_ was time-dependent: it took two values, *β*_*t*_ = *β*_*C*_ during periods of increased poultry trade, and *β*_*t*_ = *β*_0_ the remainder of the year. All other parameters, namely (i) the rate of depopulation *γ* and (ii) the rate of repopulation *δ* were assumed to be time-invariant. Other technical details of the model are provided in Supplementary Method S2.

The total number of poultry farms in Vietnam was estimated from results of the 2006 rural, agriculture and fishery census^53^. It was assumed that households keeping less than 20 birds were likely to let their animals range freely and, therefore mix with other backyard poultry of their village. For this reason, we considered each rural village as a poultry farm and the total number of poultry farms was assumed to be equal to the sum of the number of poultry farms with more than 20 birds plus the number of rural villages, i.e. 3,020,100 farms.

We estimated the likely values *β*_*C*_ and *β*_0_ of *β*_*t*_ using a simple ABC rejection algorithm^54^, with all other parameters being fixed. The algorithm was conducted as follows. We sampled values of reproduction number outside and within the period of increased poultry trade (*R*_00_ and *R*_0*C*_ respectively) from their respective uniform prior distributions: [0; 1.5] for *R*_00_ and [0; 15] for *R*_0*C*_ (the determination of these prior intervals is described in Supplementary Method S3). We converted these reproduction numbers into infectious contact rates *β*_*C*_ and *β*_0_ using the relation:

$${R}_{0t}=\frac{{\beta }_{t}N}{\gamma }$$

*γ* being the rate of depopulation of infectious farms (i.e. the rate of transition from the infectious to the removed state).

These sampled parameter values were then used as input for the model to generate a simulated time series of weekly AIOs counts. The total number of reported AIOs during the study period was too small in comparison with the number of simulated AIOs due to the limited sensitivity of the passive H5N1 surveillance system. To overcome this constraint, we randomly sampled, in each simulated time series, a number of AIOs, hereafter referred to as simulated reported AIOs, equal to the number of AIOs that were actually reported during the whole study period in the considered climatic region. The sensitivity of the surveillance was assumed to be constant over time. The distribution of simulated reported AIO waiting times was estimated, and the sampled values of *β*_*C*_ and *β*_0_ were selected to approximate the observed distribution based on the selection criteria described in Supplementary Method S3. Iterations were repeated until 1000 particles were selected.

A posterior predictive check was performed. Cumulative distributions of AIO waiting times were simulated using all parameter values from the posterior distributions. The proportion of the observed cumulative distribution of AIO waiting times contained between the maximum and minimum simulated cumulative distributions was calculated. In the same way, time series of weekly AIO incidence were simulated and their correlation with the observed AIO weekly incidence was measured in the 3 climatic regions using the Pearson correlation coefficient.

As no precise information was available on the other parameters, all possible combinations of assumed minimum and maximum values of these parameters (duration of the period during which *β*_*t*_ = *β*_*C*_, *γ* and *δ*) were used for ABC parameter selection and posterior predictive check. We considered the farm infectious period (i.e. the inverse of the rate of depopulation *γ*) was between 4 and 13 days, respectively. We considered that the period during which previously depopulated farms remained empty (i.e. the inverse of the repopulation rate *δ*) was between 15 and 45 days. The detailed selection process of these ranges of values is explained in Supplementary Method S4. In addition, we considered the period of increased poultry trade, and therefore, increased infectious contact rate (*β*_*t*_ = *β*_*C*_), extended from 7 days prior to 7 days after Lunar New Year (14 days in total), (celebrations start before and extend beyond the Lunar New Year day itself).

### Computing material

All analyses and graphical representations were produced using the R software, version 3.2.0^55^.

## Electronic supplementary material

Supplementary Information
