# Supplementary material for: Economic factors influencing zoonotic disease dynamics: demand for poultry meat and seasonal transmission of avian influenza in Vietnam
Source: Sci Rep. 2017 Jul 19;7:5905. doi: 10.1038/s41598-017-06244-6 (PMC5517570; doi:10.1038/s41598-017-06244-6)
Supplement: Supplementary file 1 — Supplementary Information [file 41598_2017_6244_MOESM1_ESM.pdf]

Economic factors influencing zoonotic disease dynamics: demand for poultry meat and seasonal transmission of avian influenza in Vietnam

Alexis Delabouglise[1,2], Marc Choisy[3,4], Thang D. Phan[5], Nicolas Antoine-Moussiaux[6], Marisa Peyre[2], Ton D. Vu[5], Dirk U. Pfeiffer[1,7], Guillaume Fournié[1]

[1] Veterinary Epidemiology, Economics and Public Health Group, Department of Production and Population Health, Royal Veterinary College, University of London, Hawkshead Lane, Hatfield, Hertfordshire, AL97TA, United Kingdom

[2] AGIRs-Animal and Integrated Risk Management Research Unit, CIRAD-Agricultural Research Center for International Development, Campus International de Baillarguet, Montpellier Cedex 5, 34398, Montpellier, France

[3] Wellcome Trust Major Overseas Programme, Oxford University Clinical Research Unit, 78 Giai Phong, Dong Da, Hanoi, Vietnam

[4] MIVEGEC, University of Montpellier, CNRS 5290, IRD 224, 911 Avenue Agropolis, 64501, Montpellier cedex 5, 34394, France

[5] Center for Interdisciplinary Research on Rural Development, Vietnam National University of Agriculture, Ngo Xuan Quang Street, Trau Quy, Gia Lam, Hanoi, Vietnam

[6] FARAH-Fundamental and Applied Research for Animals & Health, University of Liège, Avenue de Cureghem 7A-7D, Liège, 4000, Belgium

[7] School of Veterinary Medicine, City University of Hong Kong, 31 To Yuen Street, Kowloon, Hong Kong

Supplementary Method S1. Use of a finite mixture model for clustering waiting times between successive avian influenza outbreaks

Assuming that infections follow a Poisson process, mixtures of 1, 2, 3 and 4 exponential distributions are fitted to the observed distribution of waiting times. The density functions of mixtures are expressed as:

$$f(x, p_1, ..., p_n, \lambda_1, ..., \lambda_n) = \sum_j^n p_j \lambda_j e^{-\lambda_j x}$$

With

x the waiting time variable

n the number of mixture components

p_j the weight of the mixture component j i.e. the proportion of waiting times belonging to the mixture component j, with $\sum_j^n p_j = 1$

λ_j the rate parameter of the mixture component j.

An *Expectation-Maximization* algorithm is used to estimate the parameters and weights of each mixture models [1,2].

The expectation and the maximization phases of the algorithm are repeated until reaching a log-likelihood lack of progress criterion [3] defined by:

$$\left| \frac{LL_{s+1} - LL_s}{LL_s} \right| < 10^{-15}$$

LL_s: Log-Likelihood at the step s.

As the objective is to identify the mixture enabling the most adequate clustering of waiting times, the mixture model with the lowest Integrated Complete Likelihood (ICL) is selected [4,5]. ICL of fitted mixture models of 1 to 4 exponential distributions are displayed in the following table:

Region	1 component	2 components	3 components	4 components
**North**	1055	959	1043	1183
**Centre**	1095	828	889	936
**South**	1248	1040	1137	1254

An ultimate *Expectation* step is then implemented to determine the posterior probability that each observed waiting time belongs to one or the other mixture component using the *expectation* formula:

$$wij = \frac{p_j f_j(x_i)}{\sum_k p_k f_k(x_i)}$$

Where f_j is the density function of the jth component and wij is the probability that a waiting time i belongs to the distribution j, provided it belongs to the selected mixture model.

Each observed waiting time is then assigned to the mixture component to which it is most likely to belong.

References

1 Schlattmann, P. *Medical Applications of Finite Mixture Models*. 252 (Springer, 2009).

2 Wang, Y. & Wang, J. The EM algorithm for the finite mixture of exponential distribution models. *Int. J. Contemp. Math. Sci.* **9**, 57-64, doi:10.12988/ijcms.2014.312133 (2014).

3 Agha, M. & Ibrahim, M. T. Maximum likelihood estimation of mixtures of distributions. *J. R. Stat. Soc. (Serie C)* **33**, 327-332 (1984).

4 Baudry, J. P., Raftery, A. E., Celeux, G., Lo, K. & Gottardo, R. Combining Mixture Components for Clustering. *J. Comput. Graph. Stat.* **9**, 332-353, doi:10.1198/jcgs.2010.08111 (2010).

5 Biernacki, C., Celeux, G. & Govaert, G. Assessing a mixture model for clustering with the integrated completed likelihood. *IEEE Trans. Pattern Anal. Mach. Intell.* **22**, 719-725, doi:10.1109/34.865189 (2000).

Supplementary Method S2. The structure of the stochastic SIR model of inter-farm H5N1 transmission

The model is in discrete time, with a time step being equal to half a day. The number T_{S-I} of susceptible farms becoming infectious between time t and $t+\Delta t$ was generated through a binomial process with the number of susceptible farms S_t at time t as the number of trials, and the force of infection $1-e^{-\beta_t I_t \Delta t}$ as the probability of success:

$$T_{S-I} \sim Bin\left(S_t,\ 1-e^{-\beta_t I_t \Delta t}\right)$$

With β_t being the daily infectious contact rate, Δt being the time step length, and I_t the number of infectious farming units at time t. Likewise, the number of infectious farms being removed (i.e. depopulated) and the number of removed farms being repopulated between time t and $t+\Delta t$ were generated through binomial processes:

$$T_{I-R} \sim Bin\left(I_t,\ 1-e^{-\gamma \Delta t}\right)$$

With γ being the rate of depopulation, and

$$T_{R-S} \sim Bin\left(R_t,\ 1-e^{-\delta \Delta t}\right)$$

with δ being the rate of restocking and R_t the number of depopulated farms at time t.

Transmission was density-dependent. Moreover, we assumed homogeneous mixing, and ignored spatial heterogeneity in the transmission process, to ensure that the model is as parsimonious as possible while allowing exploration of the temporal variations in viral spread.

Supplementary Method S3. Characteristics of the Approximate Bayesian computation algorithm

1.Selection criteria used in the Approximate Bayesian Computation (ABC) algorithm

A proposed particle was selected if it meets the three following criteria:

1. Kolmogorov-Smirnov distance between simulated and observed cumulated distributions of waiting times between AIOs [1] needed to be lower than a pre-defined threshold $T = 0.2$. Further reduction of this threshold did not affect the shape of the posterior distribution.
2. The maximum proportion of removed farms needed to be below 25% at any time step. A higher proportion appears rather unrealistic as it means that poultry production would be majorly disrupted, which has never been observed since the first AI epizootic in 2003-2004 [2]. More importantly, it is necessary to limit the proportion of recovered farms in order for the ABC algorithm to return closed intervals of posterior particles. Indeed, for high values of β_C , a large majority of farms are infected during the period of increased poultry trade and then removed. The number of AIOs then remains very low until replenishment of the susceptible compartment (i.e. repopulation of depopulated farms). This leads to an infinite range of values of β_C being selected as outbreak time series simulated with infinitely high sampled values of β_C may still produce low Kolmogorov-Smirnov distances.
3. AIOs must be reported during the last year, i.e. the disease must be maintained throughout the study period.

2.Determination of prior intervals of R0

We first performed algorithm iterations by sampling values in a unique large prior interval [0; 30] until selecting 100 particles. As all selected particles verified $R0_0 < 1.1$ and $R0_c < 14$ we narrowed the prior intervals to [0; 1.5] and [0; 20] for sampling values of, respectively $R0_0$ and $R0_C$.

3.Resulting selection rate

With the abovementioned selection criteria and prior intervals, it took on average 60 to 4580 iterations to select a particle (i.e. a set of sampled values of β_C and β_0), depending on the values of the other fixed parameters (i.e. period during which $\beta(t) = \beta_C$, γ and δ) and the climatic region.

References

1 Wayne, D. Kolmogorov–Smirnov one-sample test in *Applied Nonparametric Statistics* 319-330 (PWS-Kent, 1990).

2 Minh, P. Q. *et al.* Spatio-temporal epidemiology of highly pathogenic avian influenza outbreaks in the two deltas of Vietnam during 2003-2007. *Prev. Vet. Med.* **89**, 16-24, doi:10.1016/j.prevetmed.2009.01.004 (2009).

Supplementary Method S4. Estimation of the interval of possible durations of farm infectious period and recovery period

1. **Infectious period**

The method used for estimating the range of possible durations of infectious period of poultry farms is similar to the one used in[1]. This method enables estimating the length of the detection period, i.e. the length of time from the infection of a flock to the detection of the infection by the farmer. The infectious period of a farm was then equal to the detection period + 1 day, as it was assumed that it took one day for a farmer to depopulate his farm (either through destruction or sale of the flock) following detection of the infection.

Model

A stochastic compartmental SEIR model was used, in which birds were either susceptible (S), infected but not yet infectious (= latently infectious) (E), infected and infectious (I) or removed (dead - All birds are assumed to die at the end of the infectious period) (R).

The model was implemented in discrete time, with hourly time steps. The number of susceptible birds moving to compartment E at each time step was generated through a binomial process with the number of susceptible farms S_t at time step *t* as the number of trials, and the force of infection $1-e^{-\beta I_t \Delta t}$ as the probability of success. The infectious contact rate was expressed as $\beta = \frac{\gamma R_0}{N}$, *N* being the initial number of birds in the farm, γ the inverse of the average duration of the infectious period and R_0 the reproduction number.

The duration of periods spent by birds in the E and I compartments were not exponentially distributed. Instead, these periods were the sum of a fixed minimum duration and an additional stochastic integer duration generated from a binomial distribution. Further details are provided in[2].

It was assumed that detection occurs when reaching a certain cumulative mortality threshold T over a 2 days' period, i.e. when

$$\frac{R_t - R_{t-2}}{N} \geq T$$

Data

The number of birds per farm was supposed to vary from 20 to 1,000[3,4]. The latent and infectious period of individual birds were taken from[5]. The latent period distribution ranged from 3 to 11 hours with a mean of 6 hours. The infectious period ranged from 43 to 55 hours with a mean of 48 hours. Estimates of intra-flock reproduction numbers were taken from[5] and[6]. The legal definition of a suspicion of H5N1 in a poultry flock in Vietnam is 5% cumulated mortality over 2 consecutive days[7]. We considered a threshold of 10% cumulated mortality in 2 consecutive days as it constitutes a more realistic assumption. It is particularly true in the case of smallholder farms with a limited number of birds which represent more than 90% of poultry farms of Viet Nam[3].

Results

For each set of parameters, the estimation of the detection period was based on 1000 simulations. The minimum and maximum estimates are presented in the following table:

Detection threshold (% cumulated mortality in 2 days)	R0	Flock size (number of birds)	Detection time (days)	
			Min	Max
10	3.4	20	3	7
		1000	6	12
	12	20	3	4
		1000	5	7

2. Recovery period

As no precise data was available, the length of the recovery period was estimated based on the authors' knowledge of Vietnamese poultry production. It was assumed that the period during which farms remained depopulated was unlikely to be lower than 15 days, as farmers feared re-infection caused by virus survival in the environment, but it was very unlikely to be higher than 45 days, as farmers tended to resume their production as quickly as possible for economic reasons.

References

1 Bos, M. E. *et al.* Estimating the day of highly pathogenic avian influenza (H7N7) virus introduction into a poultry flock based on mortality data. *Vet. Res.* **38**, 493-504, doi:10.1051/vetres:2007008 (2007).

2 Fournie, G., Guitian, F. J., Mangtani, P. & Ghani, A. C. Impact of the implementation of rest days in live bird markets on the dynamics of H5N1 highly pathogenic avian influenza. *J R Soc Interface* **8**, 1079-1089, doi:10.1098/rsif.2010.0510 (2011).

3 General Statistics Office of Vietnam. *Results of the 2006 Rural, Agriculture and Fishery Census*. (Vietnam Statistical Publishing House, 2007).

4 General Statistics Office of Vietnam. *Results of the 2011 Rural, Agriculture and Fishery Census*. (Vietnam Statistical publishing House, 2012).

5 Bouma, A. *et al.* Estimation of transmission parameters of H5N1 avian influenza virus in chickens. *Plos Pathog* **5**, e1000281, doi:10.1371/journal.ppat.1000281 (2009).

6 Poetri, O. N. *et al.* An inactivated H5N2 vaccine reduces transmission of highly pathogenic H5N1 avian influenza virus among native chickens. *Vaccine* **27**, 2864-2869, doi:10.1016/j.vaccine.2009.02.085 (2009).

7 Department of Animal Health of Vietnam. *Official Guide of avian influenza surveillance in years 2011-2012*. (Department of Animal Health of Vietnam, 2011).

Economic factors influencing zoonotic disease dynamics: demand for poultry meat and seasonal transmission of avian influenza in Vietnam

Alexis Delabouglise[1,2], Marc Choisy[3,4], Thang D. Phan[5], Nicolas Antoine-Moussiaux[6], Marisa Peyre[2], Ton D. Vu[5], Dirk U. Pfeiffer[1,7], Guillaume Fournié[1]

[1] Veterinary Epidemiology, Economics and Public Health Group, Department of Production and Population Health, Royal Veterinary College, University of London, Hawkshead Lane, Hatfield, Hertfordshire, AL97TA, United Kingdom

[2] AGIRs-Animal and Integrated Risk Management Research Unit, CIRAD-Agricultural Research Center for International Development, Campus International de Baillarguet, Montpellier Cedex 5, 34398, Montpellier, France

[3] Wellcome Trust Major Overseas Programme, Oxford University Clinical Research Unit, 78 Giai Phong, Dong Da, Hanoi, Vietnam

[4] MIVEGEC, University of Montpellier, CNRS 5290, IRD 224, 911 Avenue Agropolis, 64501, Montpellier cedex 5, 34394, France

[5] Center for Interdisciplinary Research on Rural Development, Vietnam National University of Agriculture, Ngo Xuan Quang Street, Trau Quy, Gia Lam, Hanoi, Vietnam

[6] FARAH-Fundamental and Applied Research for Animals & Health, University of Liège, Avenue de Cureghem 7A-7D, Liège, 4000, Belgium

[7] School of Veterinary Medicine, City University of Hong Kong, 31 To Yuen Street, Kowloon, Hong Kong

Supplementary Figure S1

Results of wavelet coherence analysis between time series of reported avian influenza outbreaks and climate variables in the 3 identified climatic regions of Vietnam. Above: North, middle: Centre, below: South**.** Left: Wavelet coherence indicated by a colour spectrum (blue: weak coherence, red: high coherence) as a function of the month of study period (x-axis) and the wavelet period (i.e. inverse frequency of wavelet oscillations) (y-axis). Black lines delineate areas of significant coherence between wavelet transforms (with an alpha risk ≤ 5%). White lines delineate the cone of influence, i.e the area where computed coherences are strongly influenced by the edge effects. Right: identified phase shifts from wavelet transforms of AI incidence to wavelet transforms of the climatic variable, when the two are significantly coherent.

Absolute humidity

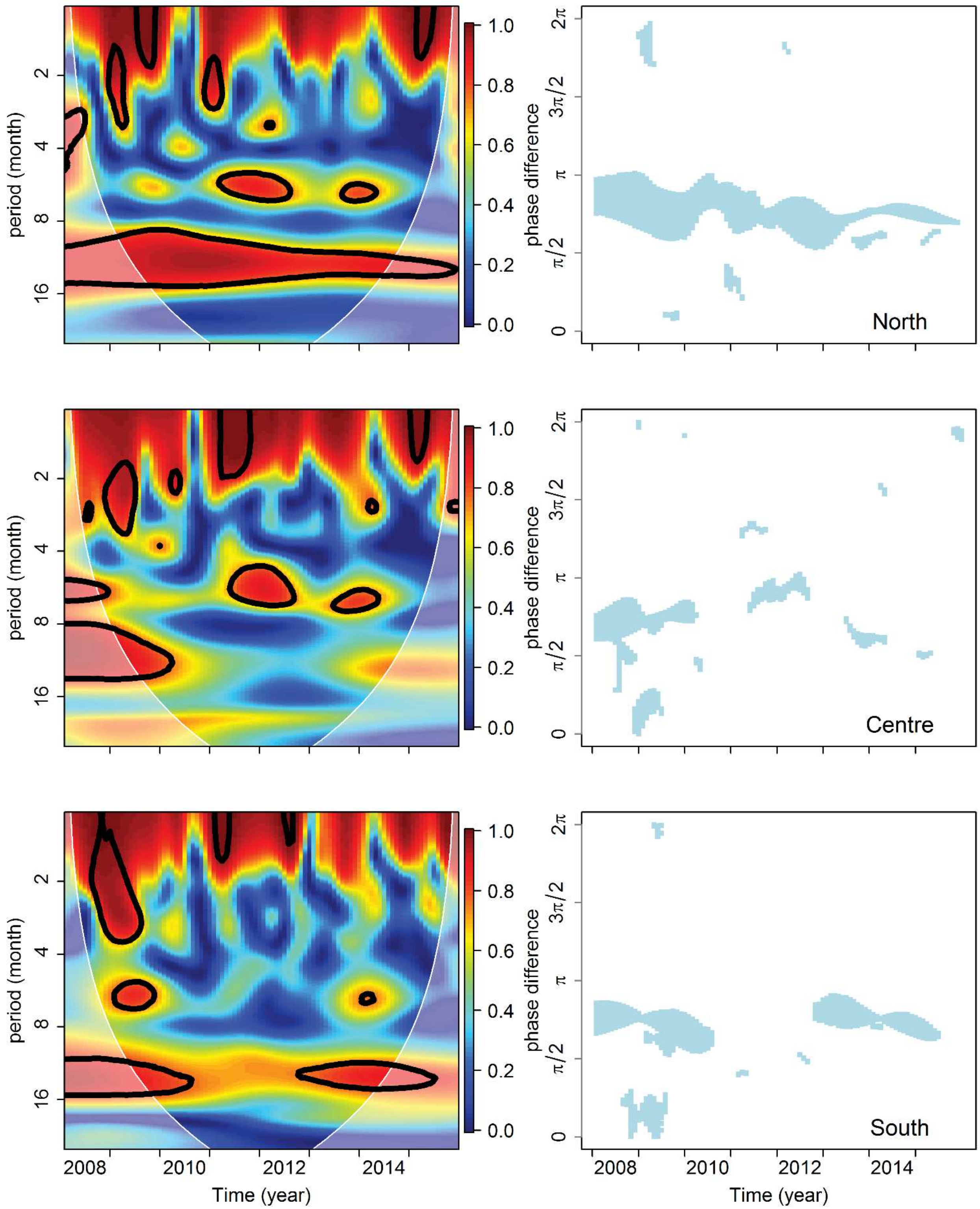

Relative humidity

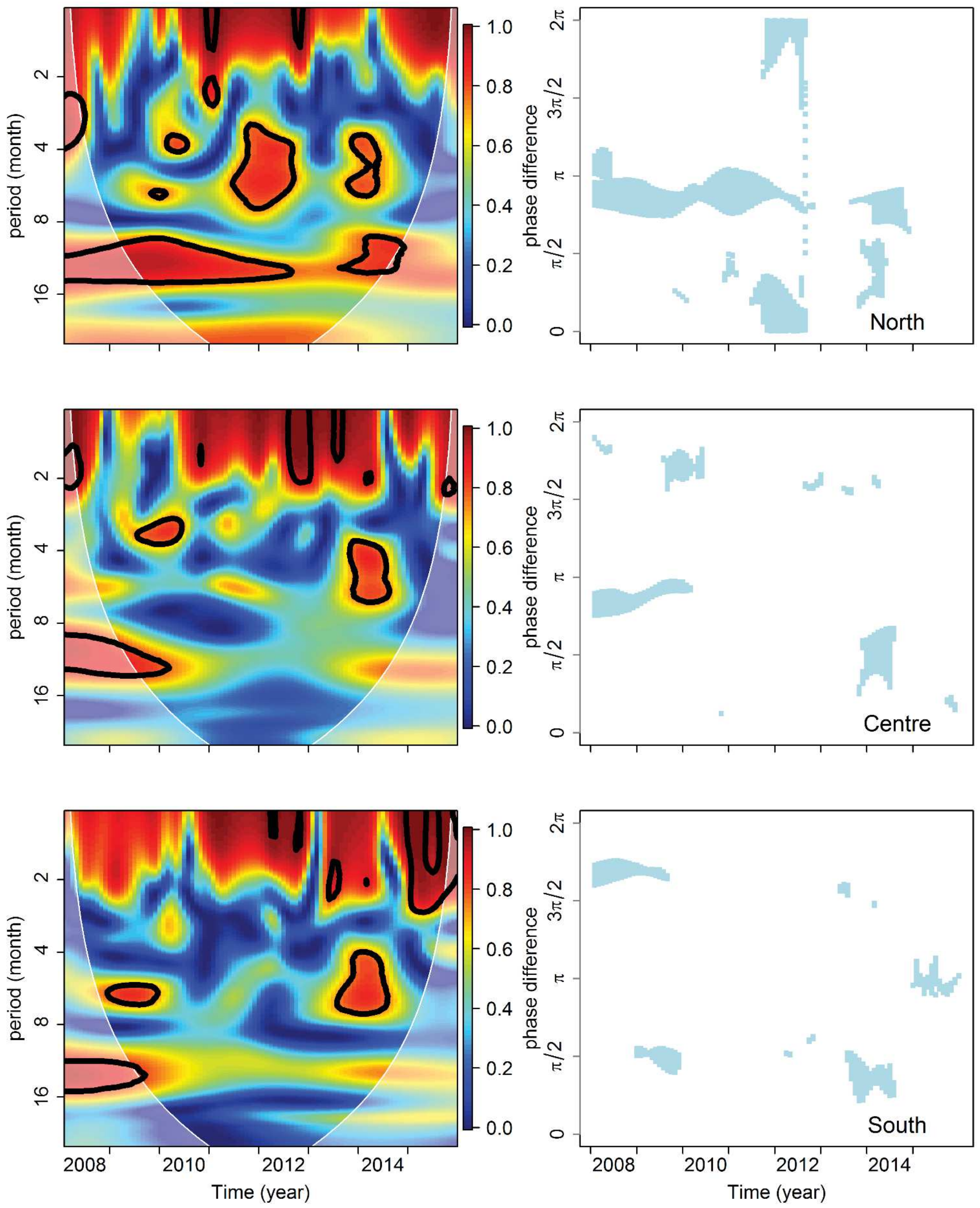

Rainfall

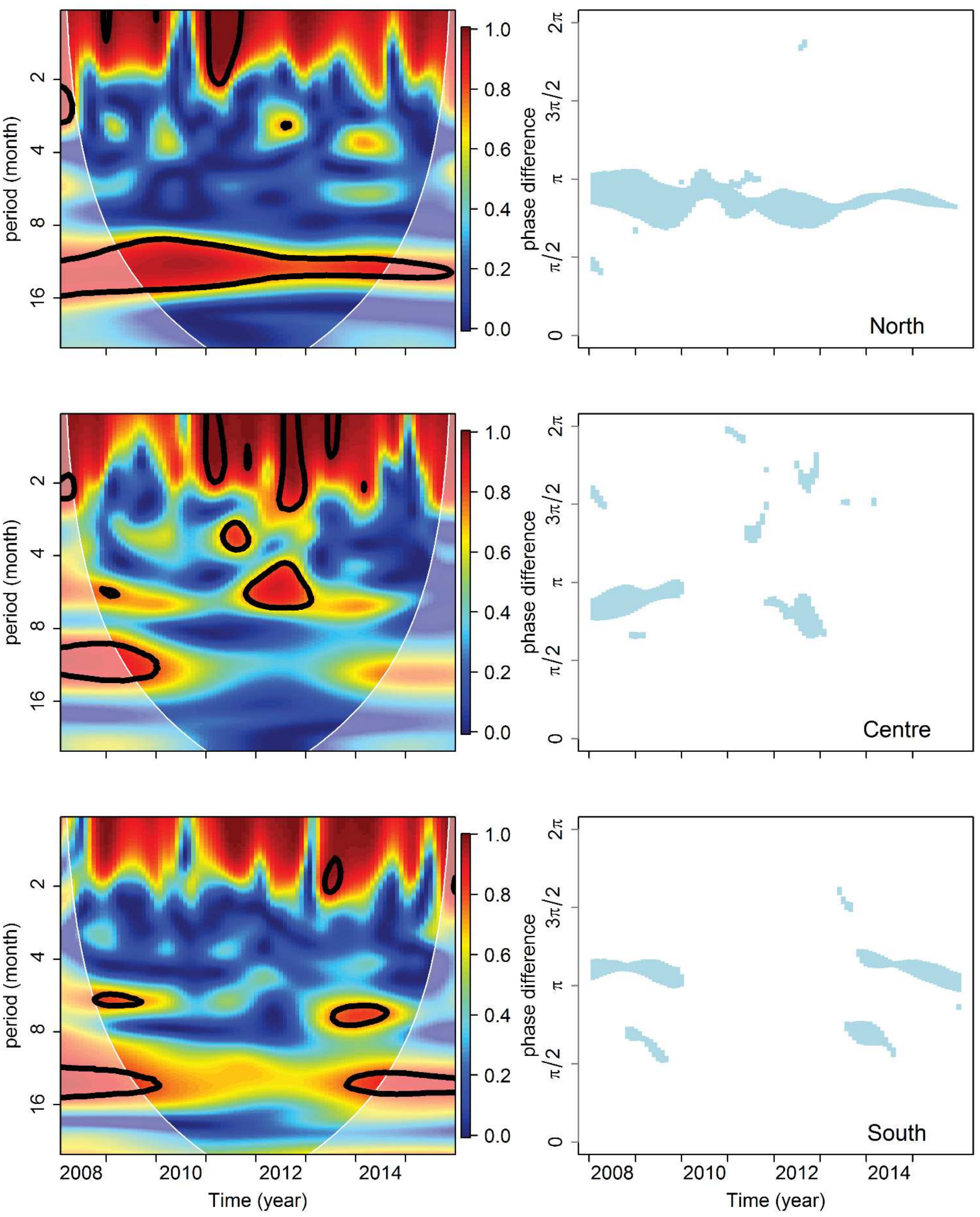

period (month)
2
4
8
16
1.0
0.8
0.6
0.4
0.2
0.0
phase difference
0
π/2
π
3π/2
2π
North
Centre
South
2008
2010
2012
2014
Time (year)

Average temperature

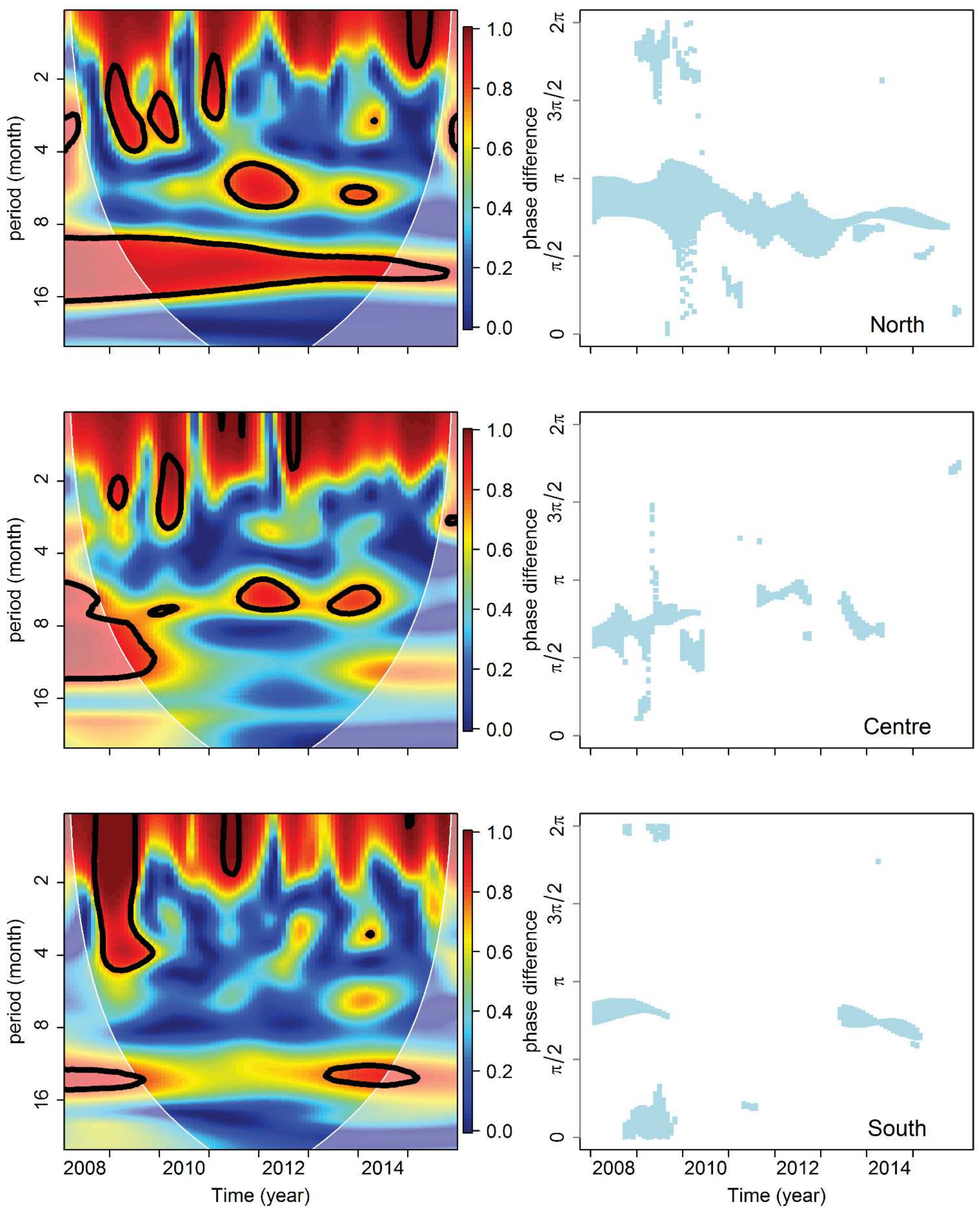

Sunshine duration

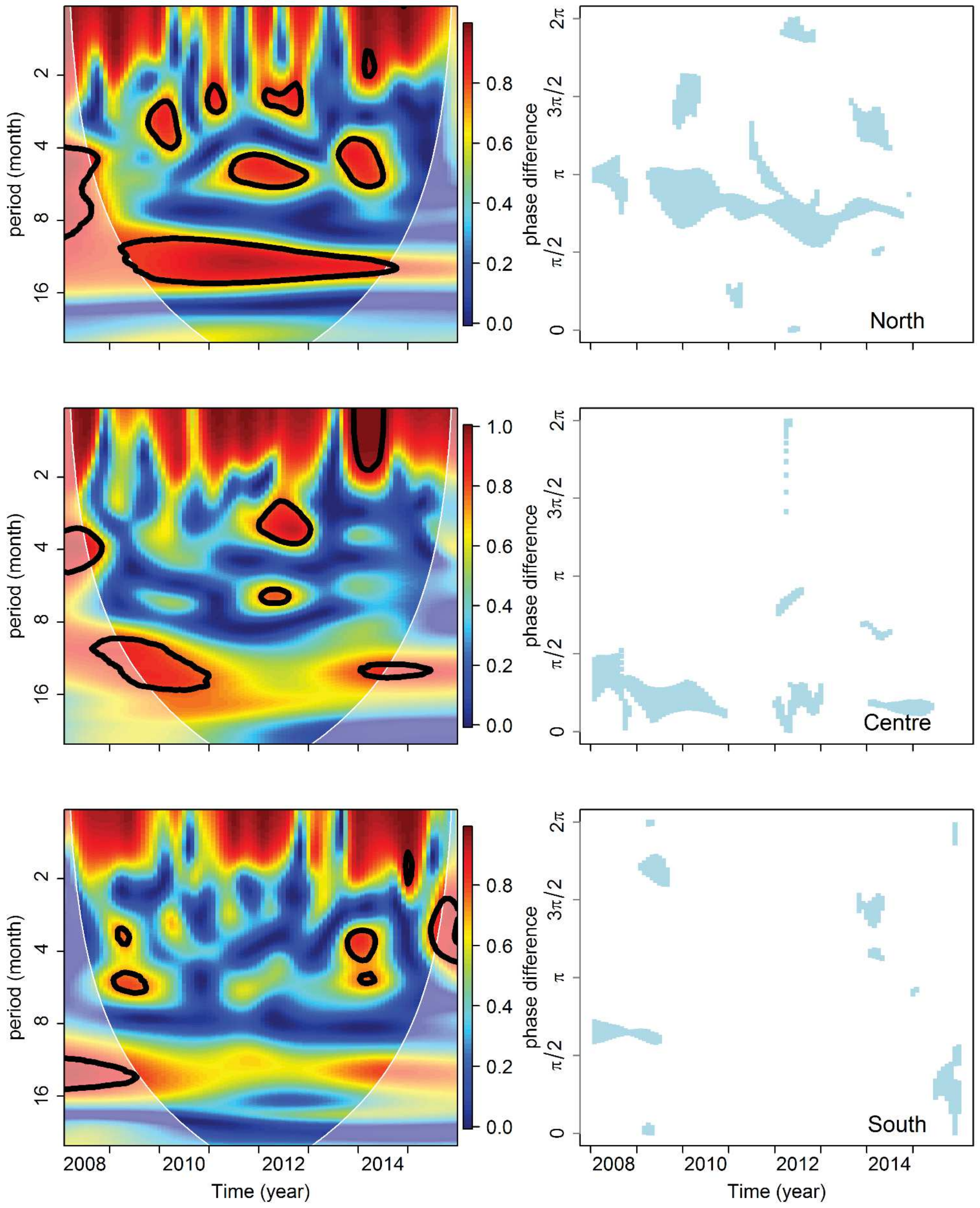

Supplementary Figure S2

Estimated increase in infectious contact rates during periods of increased poultry meat consumption and posterior predictive checks. (A) Distribution of posterior ratios of infectious contact rates (β_c/β_0). (B) Simulated and reported cumulative distributions of waiting times between successive avian influenza outbreaks. (C) Weekly incidence of reported and simulated avian influenza outbreaks across study period. Simulations are based on selected particles.

Region: North

Duration of infectious period: 4 days

Time before repopulation: 15 days Duration

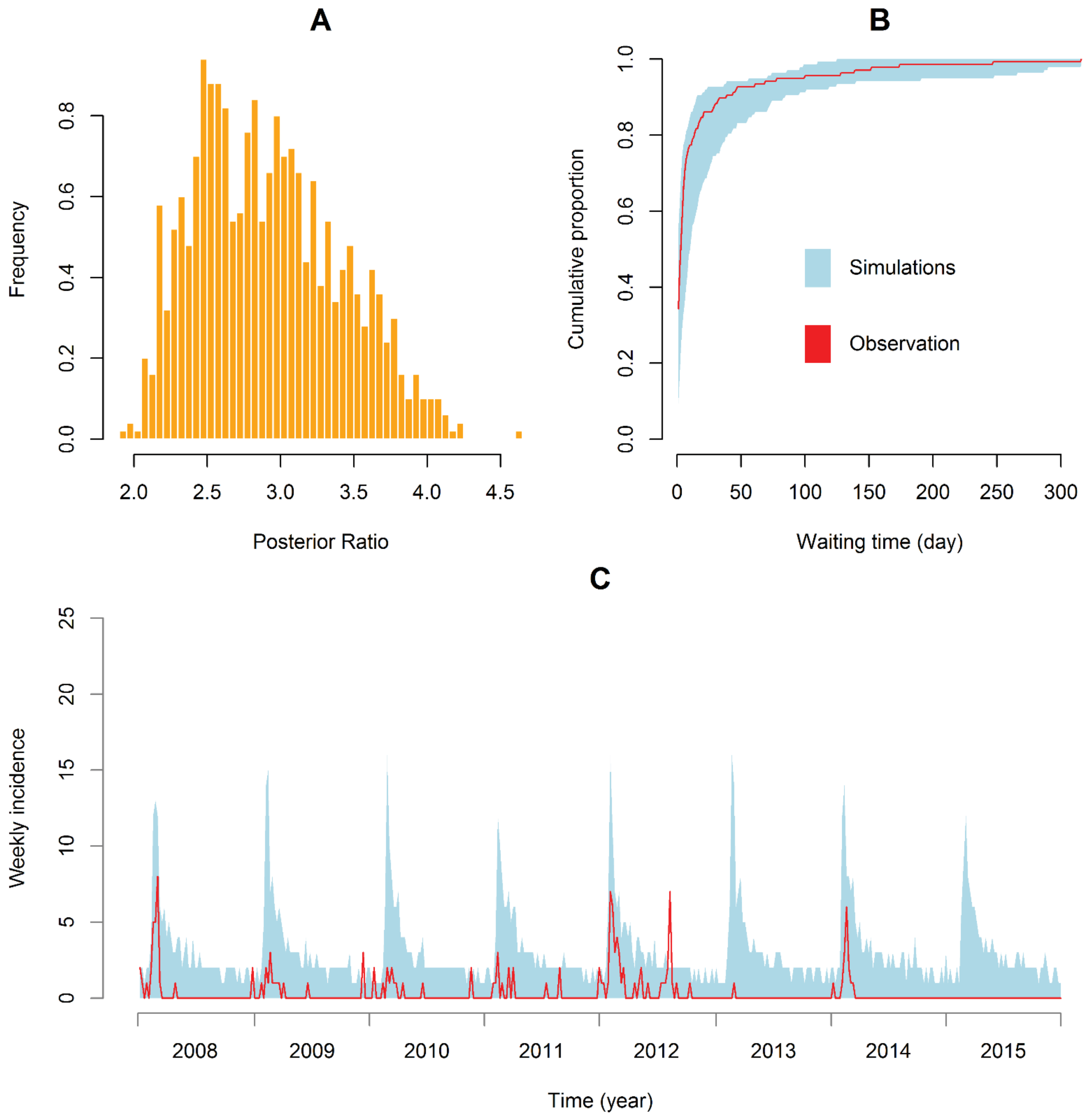

Region: Centre

Duration of infectious period: 4 days

Time before repopulation: 15 days Duration

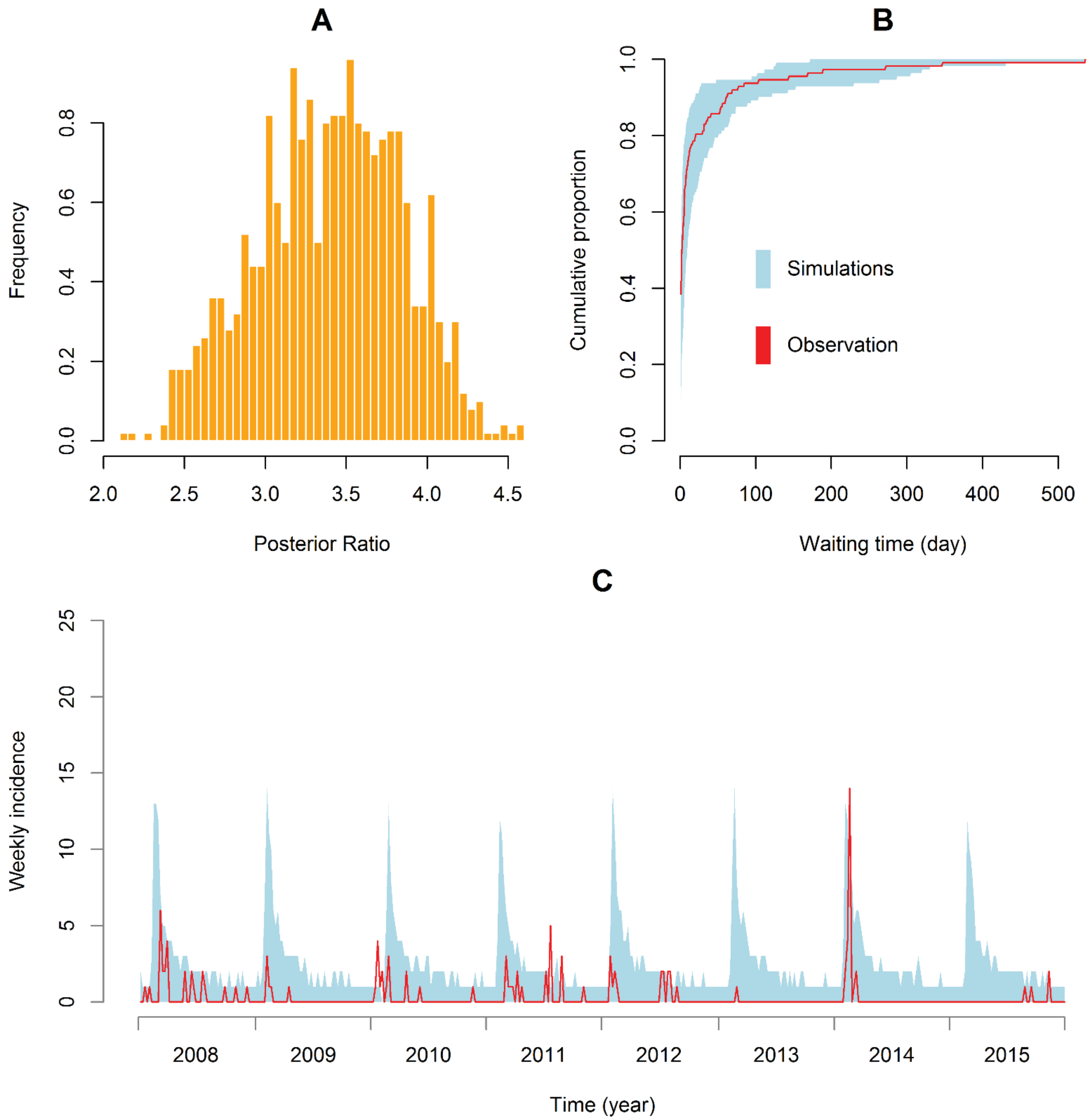

Region: South

Duration of infectious period: 4 days

Time before repopulation: 15 days Duration

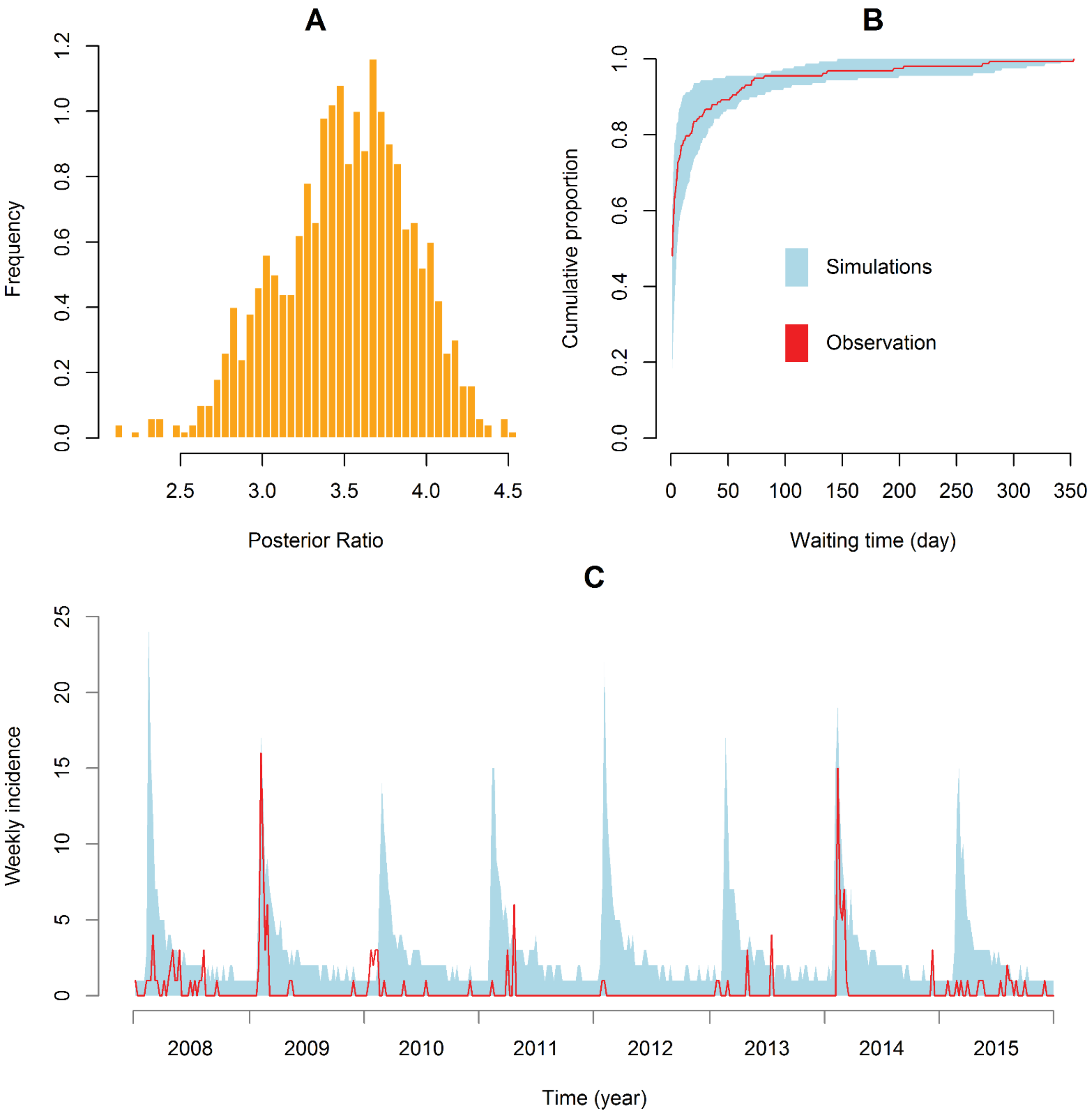

Region: North

Duration of infectious period: 4 days

Time before repopulation: 45 days Duration

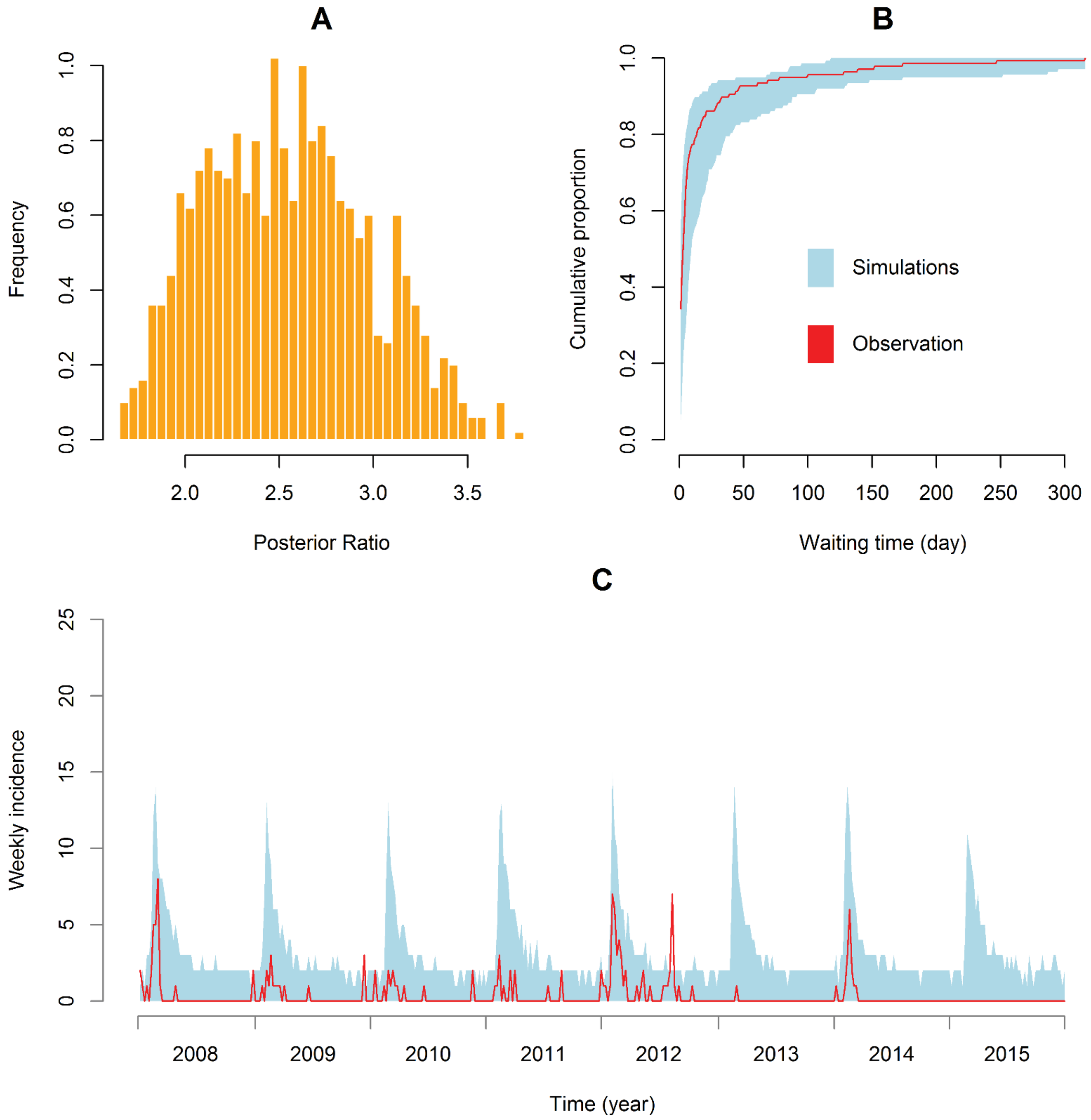

Region: Centre

Duration of infectious period: 4 days

Time before repopulation: 45 days Duration

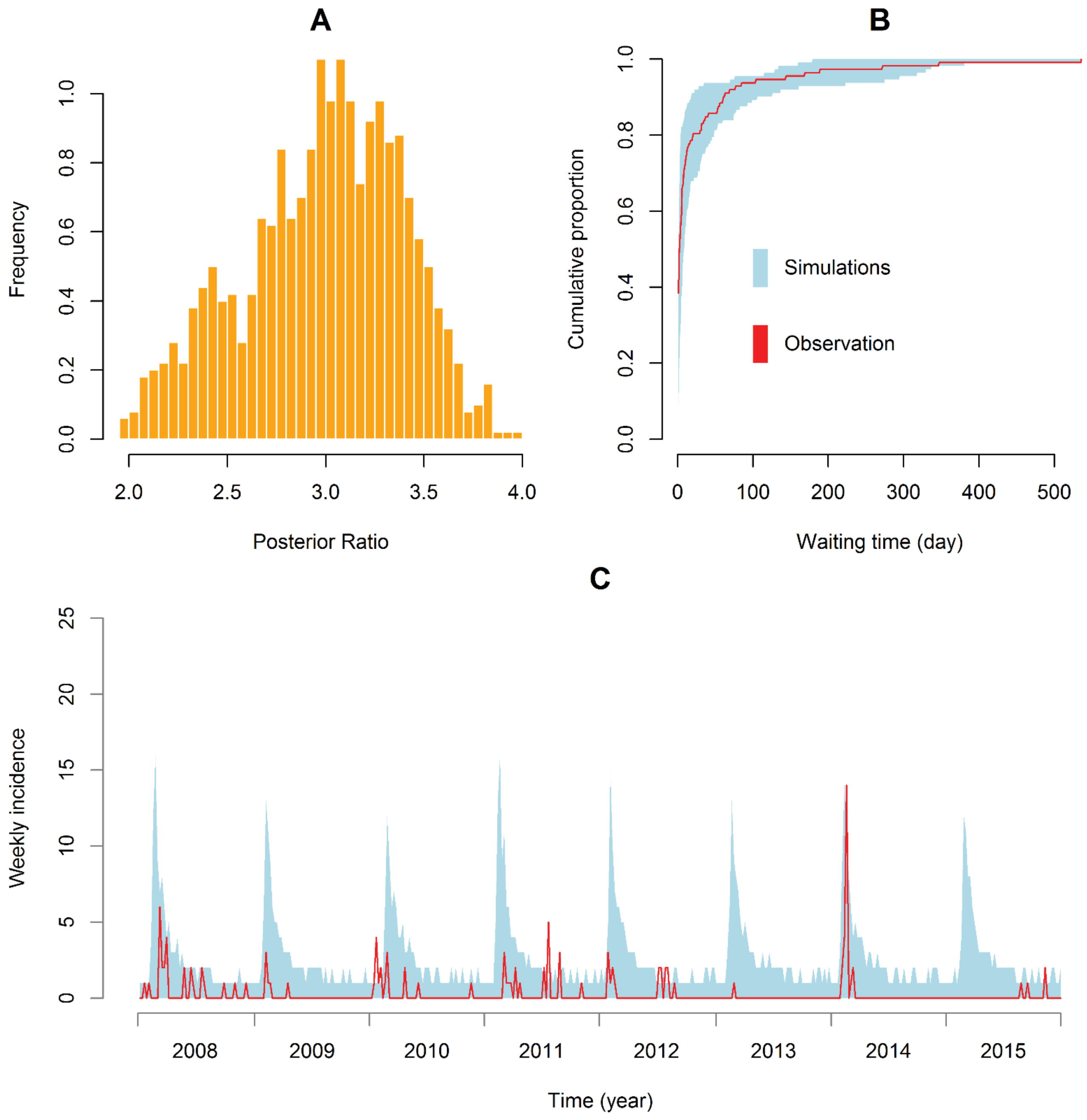

Region: South

Duration of infectious period: 4 days

Time before repopulation: 45 days Duration

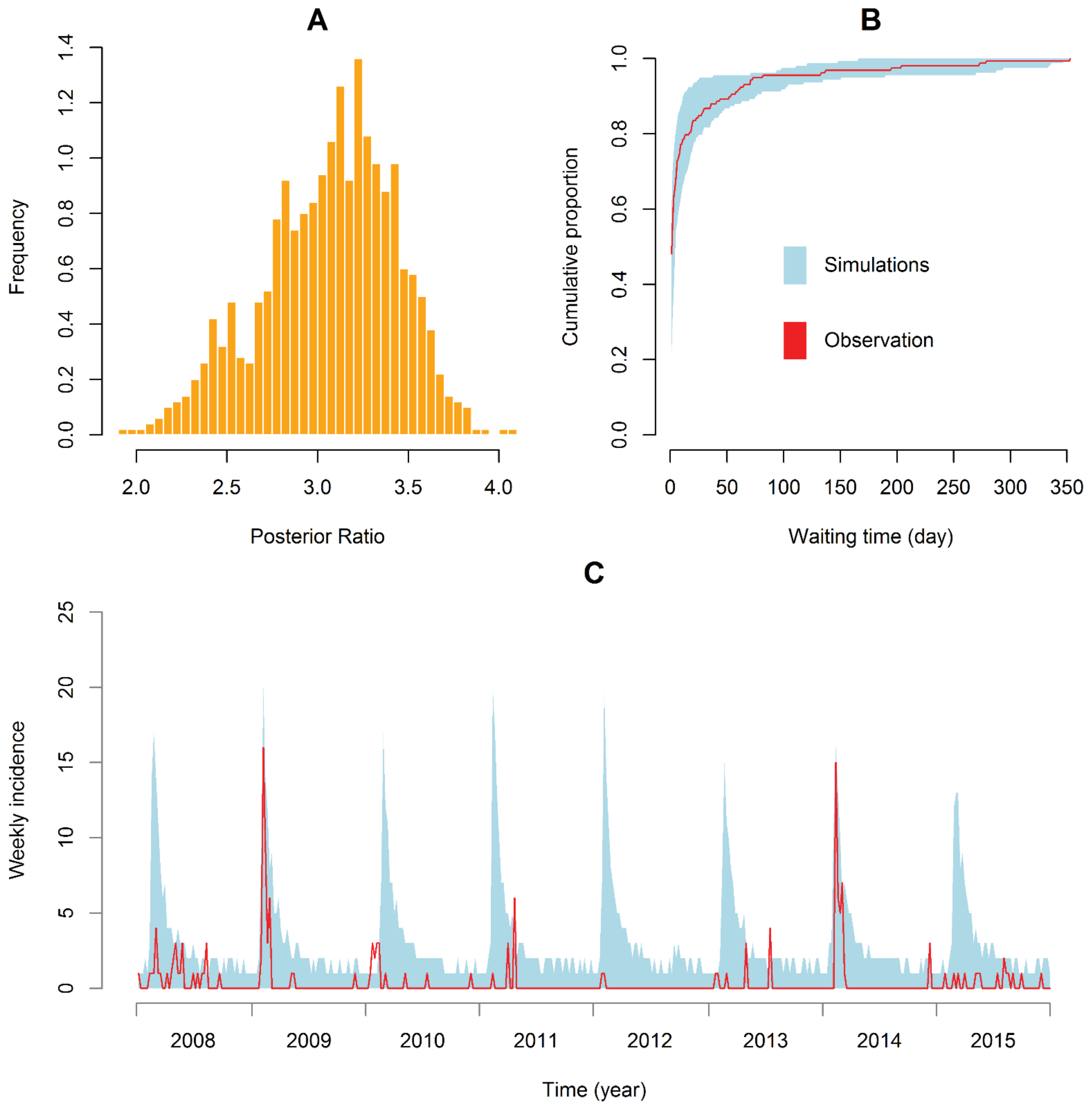

Region: North

Duration of infectious period: 13 days

Time before repopulation: 15 days Duration

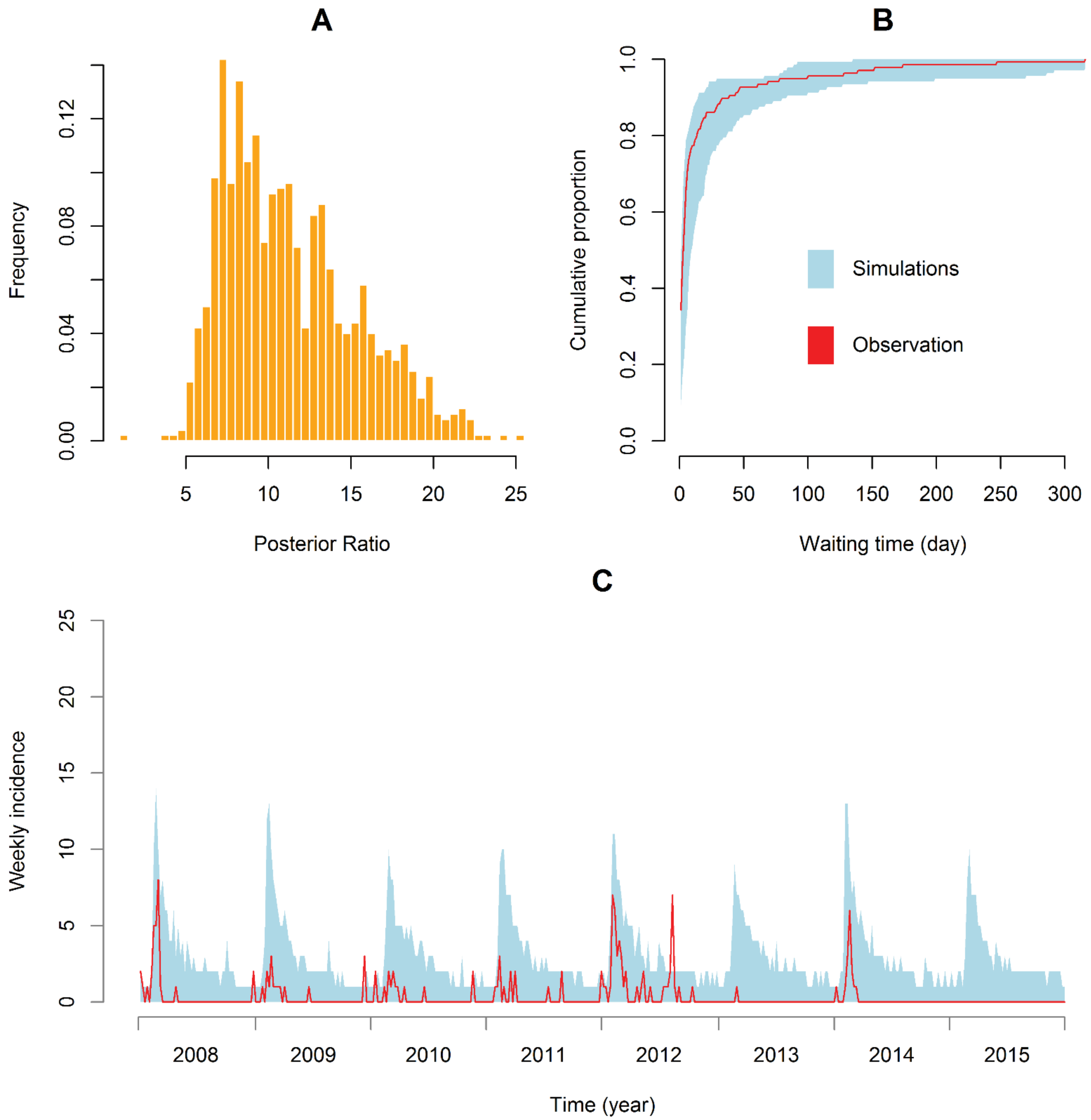

Region: Centre

Duration of infectious period: 13 days

Time before repopulation: 15 days Duration

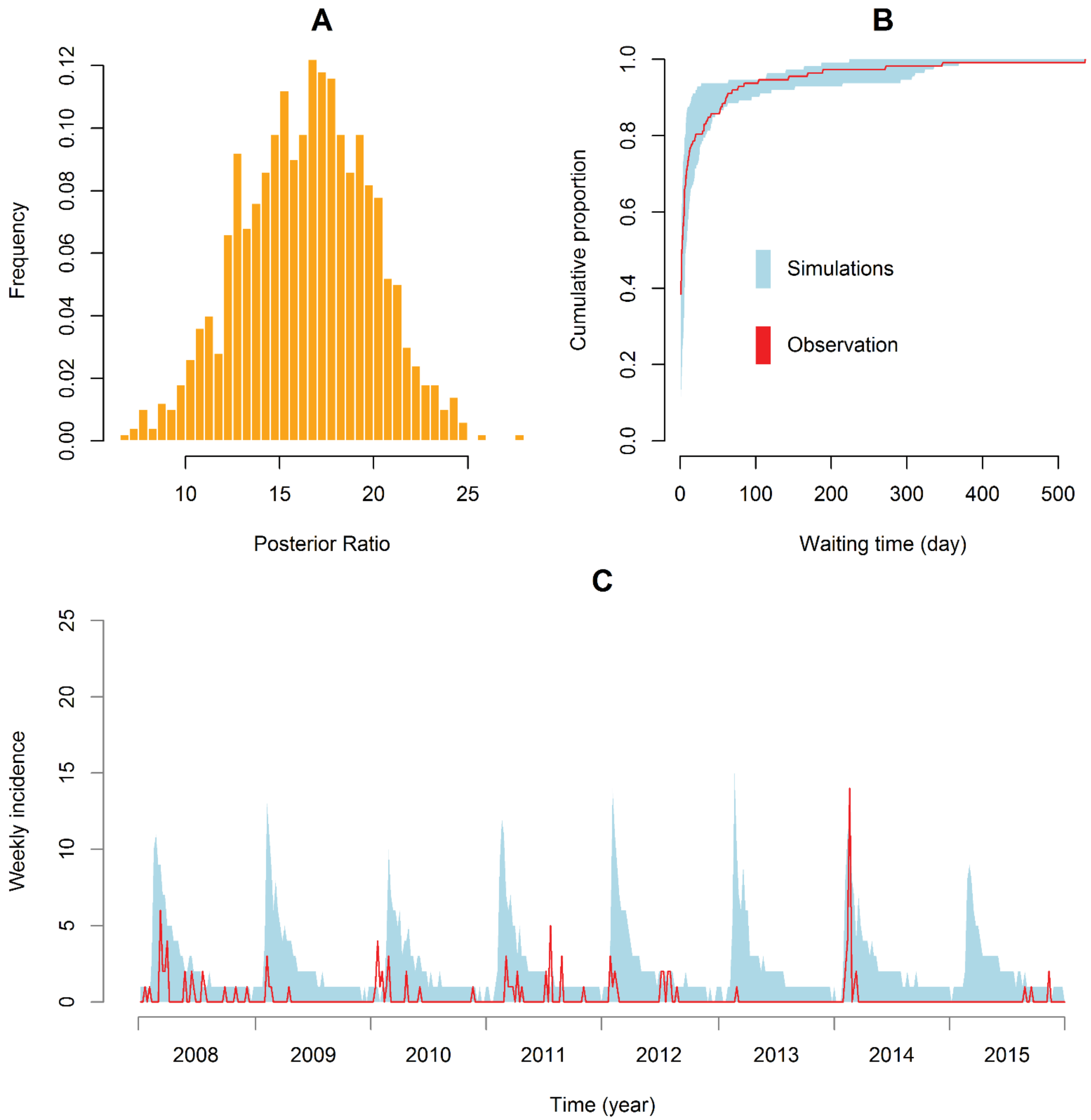

Region: South

Duration of infectious period: 13 days

Time before repopulation: 15 days Duration

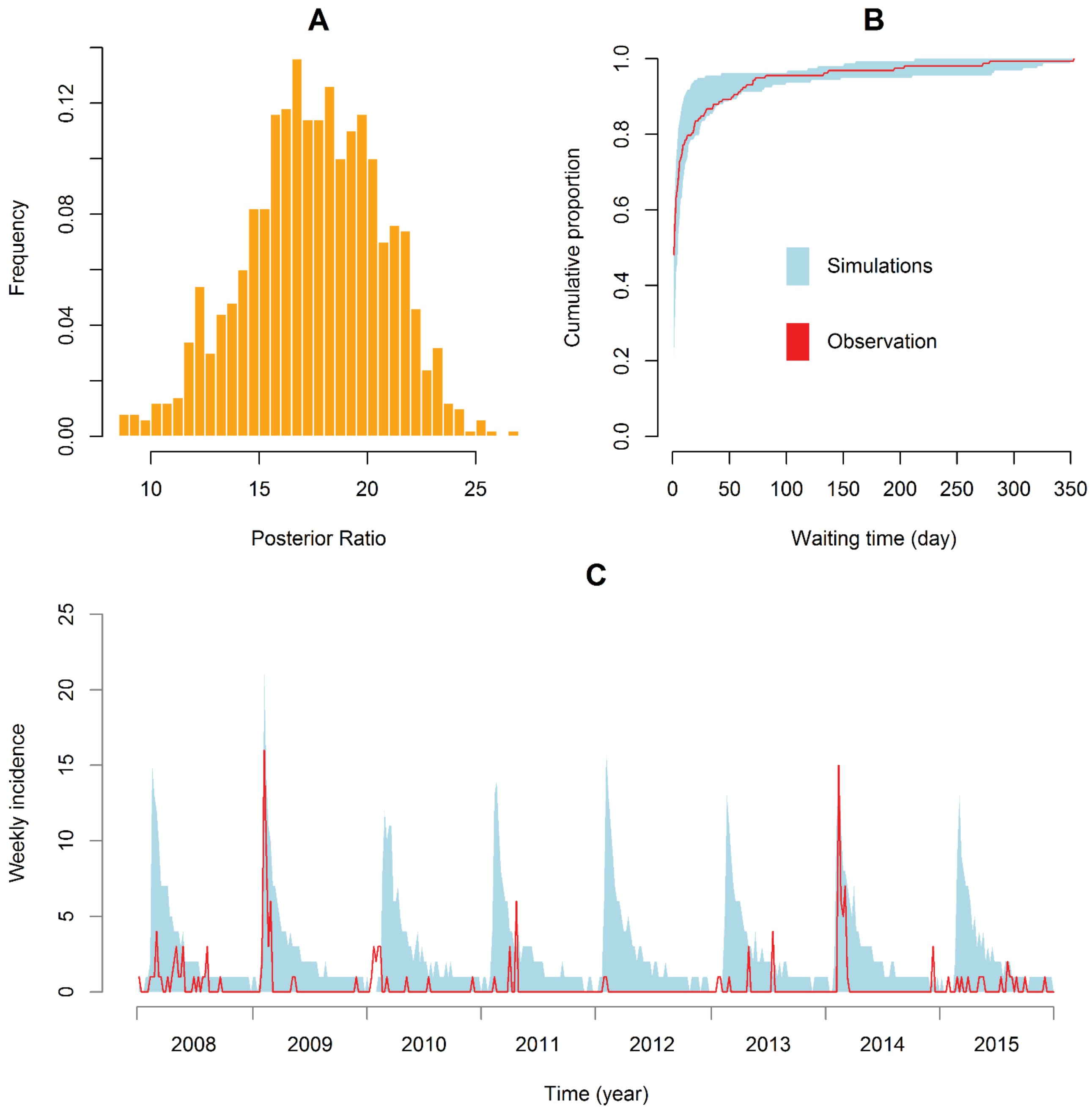

Region: North

Duration of infectious period: 13 days

Time before repopulation: 45 days Duration

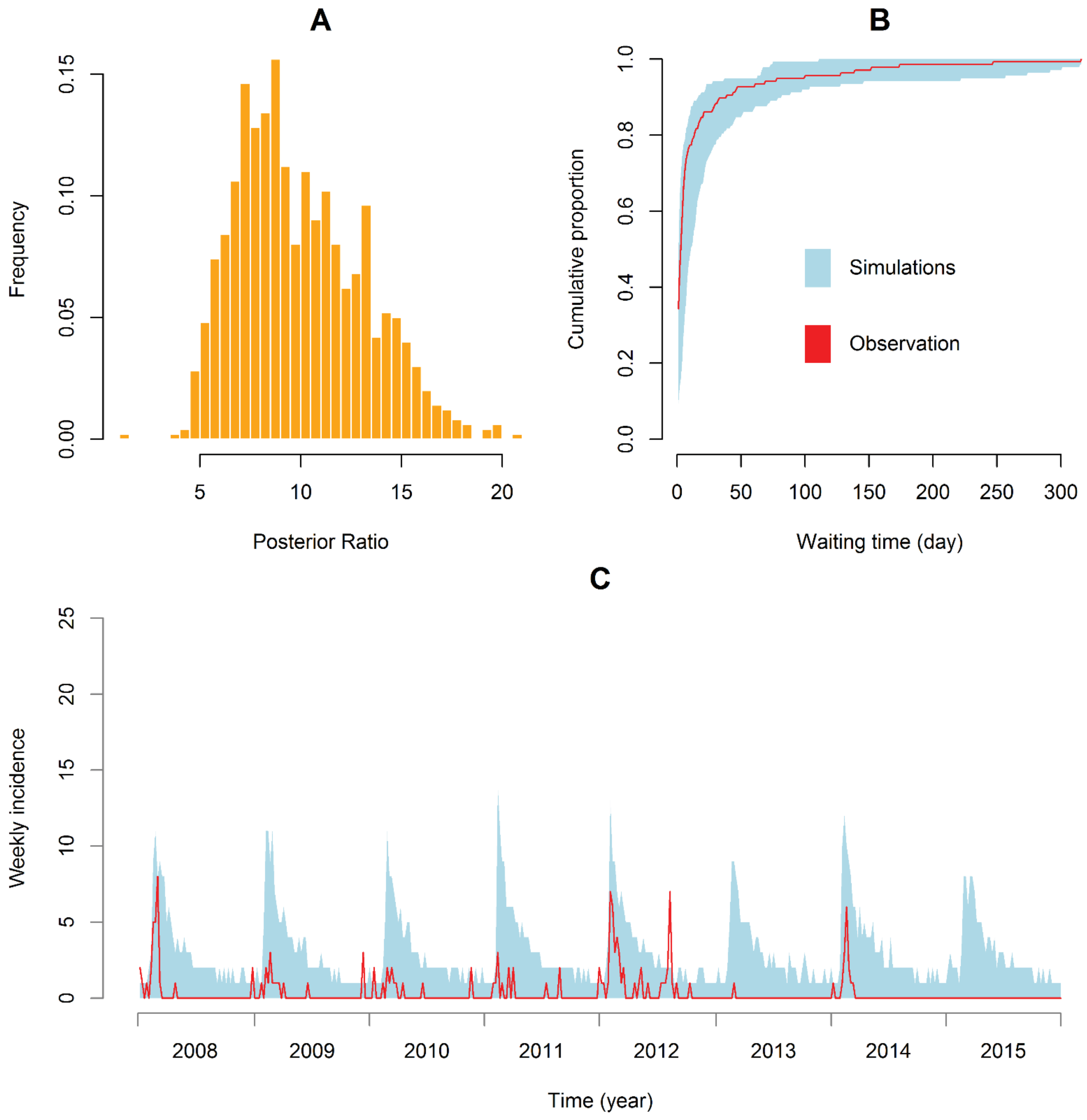

Region: Centre

Duration of infectious period: 13 days

Time before repopulation: 45 days Duration

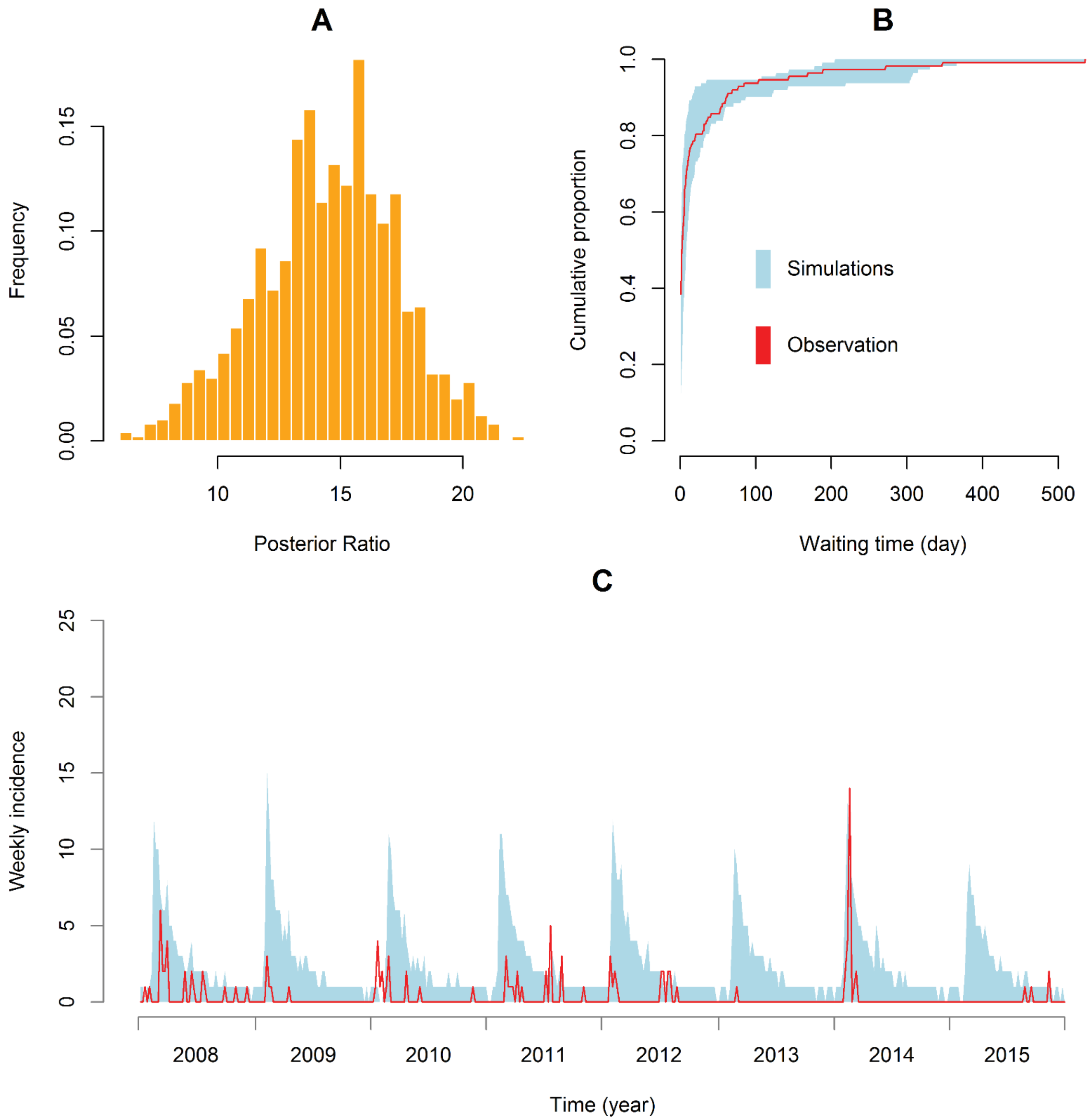

Region: South

Duration of infectious period: 13 days

Time before repopulation: 45 days Duration

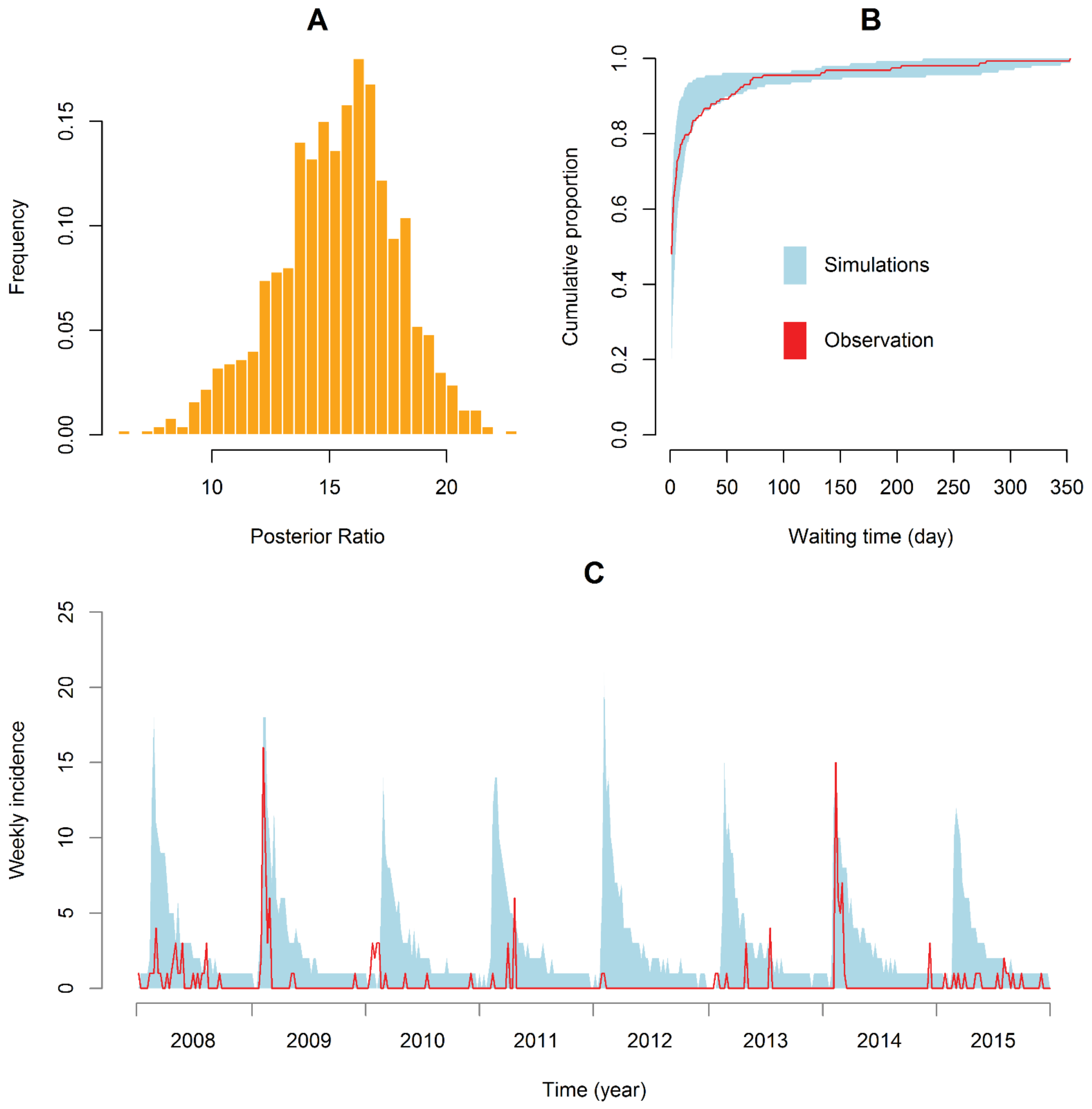

Economic factors influencing zoonotic disease dynamics: demand for poultry meat and seasonal transmission of avian influenza in Vietnam

Alexis Delabouglise[1,2], Marc Choisy[3,4], Thang D. Phan[5], Nicolas Antoine-Moussiaux[6], Marisa Peyre[2], Ton D. Vu[5], Dirk U. Pfeiffer[1,7], Guillaume Fournié[1]

[1] Veterinary Epidemiology, Economics and Public Health Group, Department of Production and Population Health, Royal Veterinary College, University of London, Hawkshead Lane, Hatfield, Hertfordshire, AL97TA, United Kingdom

[2] AGIRs-Animal and Integrated Risk Management Research Unit, CIRAD-Agricultural Research Center for International Development, Campus International de Baillarguet, Montpellier Cedex 5, 34398, Montpellier, France

[3] Wellcome Trust Major Overseas Programme, Oxford University Clinical Research Unit, 78 Giai Phong, Dong Da, Hanoi, Vietnam

[4] MIVEGEC, University of Montpellier, CNRS 5290, IRD 224, 911 Avenue Agropolis, 64501, Montpellier cedex 5, 34394, France

[5] Center for Interdisciplinary Research on Rural Development, Vietnam National University of Agriculture, Ngo Xuan Quang Street, Trau Quy, Gia Lam, Hanoi, Vietnam

[6] FARAH-Fundamental and Applied Research for Animals & Health, University of Liège, Avenue de Cureghem 7A-7D, Liège, 4000, Belgium

[7] School of Veterinary Medicine, City University of Hong Kong, 31 To Yuen Street, Kowloon, Hong Kong

Supplementary Table S1

Values of posterior rates of infectious contacts during and outside the defined at-risk period and their ratio selected through Approximate Bayesian Computation in the three pre-defined climatic regions of Vietnam (Median, minimum and maximum).

**Duration of infectious period (days)***	**Duration of recovery period (days)****	**Region**	β_0 $(\times 10^{-8})$	β_C $(\times 10^{-8})$	β_C/β_0
4	15	North	7.9 (7.6-8.4)	22.7 (16-35.3)	2.9 (1.9-4.6)
4	15	Centre	7.8 (7.5-8.3)	26.7 (17.9-34.8)	3.4 (2.1-4.6)
4	15	South	7.8 (7.5-8.4)	27.5 (17.9-34.5)	3.5 (2.1-4.5)
4	45	North	8.1 (7.7-8.9)	20.5 (14-29.7)	2.5 (1.7-3.8)
4	45	Centre	8 (7.7-8.6)	24.3 (17-30.8)	3 (2-4)
4	45	South	8 (7.7-8.6)	24.7 (16.8-31.6)	3.1 (1.9-4.1)
13	15	North	2 (1.5-2.6)	20.8 (2.7-36.8)	10.6 (1-25.3)
13	15	Centre	1.7 (1.4-2.3)	28.4 (15.2-40)	16.6 (6.6-27.8)
13	15	South	1.7 (1.4-2.1)	29.4 (18.2-38.2)	17.6 (8.5-26.6)
13	45	North	2 (1.5-2.6)	19 (2.7-33.4)	9.4 (1-20.7)
13	45	Centre	1.8 (1.5-2.3)	26.1 (14.1-35.1)	14.7 (6.3-22.3)
13	45	South	1.7 (1.5-2.2)	27.1 (13.6-35.2)	15.5 (6.5-23)

*Period from the infection of farm birds to farm clearing

**Period from farm clearing to repopulation
